# Surgical Outcomes of Transperitoneal Para-Aortic Lymphadenectomy Compared With Extraperitoneal Approach in Gynecologic Cancers: A Systematic Review and Meta-Analysis

**DOI:** 10.3389/fsurg.2021.779372

**Published:** 2021-12-21

**Authors:** Kun-peng Li, Xian-zhong Deng, Tao Wu

**Affiliations:** Department of Urology, Affiliated Hospital of North Sichuan Medical College, Nanchong, China

**Keywords:** extraperitoneal approach, transperitoneal approach, gynecologic cancers, systematic review, meta-analysis

## Abstract

**Purpose:** The optimal surgical approach for para-aortic lymphadenectomy (PALND) in gynecologic cancers using minimally invasive surgery (laparoscopy or robotic-assisted) is controversial. This study summarizes the current evidence on the extraperitoneal (EP) approach and compares its perioperative, surgical outcomes, and complications to the transperitoneal (TP) approach in an updated meta-analysis.

**Methods:** We performed a systematic search in PubMed, Embase, Web of Science, Cochrane Library database for randomized controlled trials (RCTs) and non-RCTs that compare EP to TP for PALND. The main outcomes included surgical, perioperative outcomes, and complications. The weighted mean difference (WMD) and odds ratio (OR) were applied for the comparison of continuous and dichotomous variables with 95% CIs. Three RCTs and 10 non-RCTs trials, including 2,354 patients were identified and enrolled in the meta-analysis.

**Results:** A total of three RCTs and ten non-RCTs trials, including 2,354 patients were identified and enrolled in the meta-analysis. We reported similar results for EP and TP in terms of the hospital stay, estimated blood loss, blood transfusion, conversion to laparotomy, total operative time, and postoperative complications (Clavien grade ≥ 1 and Clavien grade ≥ 3). However, the PALND operative time (WMD −10.46 min, 95% CI −19.04, −1.88; *p* = 0.02) and intraoperative complications (OR 0.40, 95% CI 0.23, 0.69; *p* = 0.001) were less with EP. Also, more nodes were removed in EP compared with the TP (WMD 1.45, 95% CI 0.05, 2.86; *p* = 0.04).

**Conclusions:** The EP approach did not show differences regarding surgical and perioperative parameters compared with the TP approach. However, the number of aortic nodes retrieved was higher. Furthermore, The PALND operative time and intraoperative complications were less in EP.

## Introduction

Para-aortic lymphadenectomy (PALND) is a routine method for staging or treatment of gynecological malignancies (1). In the cervical cancers and advanced vaginal cancers, PALND is to guide the fields of radiation and also for the therapeutic benefits (1, 2). In endometrial and adnexal cancer, PALND is also performed for therapeutic purposes (3). In other words, PALND is useful for tailoring the optimally therapeutic purposes of patients according to their stage, providing adjuvant treatment for patients with infiltrated lymph nodes, and eliminating recurrence risk (4).

In the past, PALND was systematically performed using laparotomy. However, extraperitoneal (EP) and transperitoneal (TP) laparoscopy have also been used to perform PALND in the last few decades (5, 6). Furthermore, some recent studies also reported that minimally invasive salvage lymphadenectomy is an effective therapeutic approach for the recurrent patients with gynecological cancer bearing lymph-node recurrence. Without affecting the prognosis of the cancer, salvage lymphadenectomy is beneficial for perioperative and postoperative outcomes (7, 8). Since the reports on the effectiveness and safety of the robot-assisted PALND in gynecologic oncology were published (9, 10), minimally invasive surgery (laparoscopic surgery or robot-assisted) has become popular among surgeons in the field of PALND (11). However, to date, the optimal surgical approach for PALND in the gynecologic cancers is still controversial. Notably, Prodromidou et al. compared the outcomes of the EP and TP in 608 patients who underwent laparoscopic lymphadenectomy and conducted a meta-analysis that demonstrated clinically equivalent results between EP and TP (12). However, the outcomes of this meta-analysis are incomplete. There is no meta-analysis comparing the overall complications, major complications, and conversion to laparotomy between EP and TP. Furthermore, the included studies are all retrospective with low quality and are not up to date. So, more studies comparing EP with TP should be added to the scientific literature including RCTs and robotic assisted.

Therefore, we conducted a state-of-the-art meta-analysis to integrate all the accumulated evidence to date.

## Methods

This systematic review and meta-analysis were conducted per the Preferred Reporting Items for Systematic Reviews and Meta-Analyses (PRISMA) statement (13).

### Literature Search Strategy, Study Selection, and Data Collection

We conducted a comprehensive electronic literature search in July 2021 in PubMed, Embase, Web of Science, and Cochrane Library database. Intervention and patient-related search terms were combined to build the following search string: laparoscopic or robot-assisted or minimally invasive and lymphadenectomy or gynecologic cancers or gynecological malignancies and transperitoneal or extraperitoneal or retroperitoneal.

Inclusion criteria were defined using the PICOS approach. P (patients): All the patients underwent EP vs. TP for staging or treating gynecological malignancies. The malignancies included cancer of the endometrium, ovary, vagina, and cervix; I (intervention): undergoing EP; C (comparator): TP was performed as a comparator; O (outcome): one or more of the following outcomes: perioperative outcomes, surgical outcomes, and complications; S (study type): a retrospective, prospective comparative studies or RCTs. Exclusion criteria: (1) non-comparative studies; (2) editorial comments, meeting abstracts, case reports, or letters to the editor; and (3) none of the defined outcome measures analysis.

A total of two independent reviewers extracted the data. The following data were extracted for each study: (1) General information related to the manuscript: first author, year of publication, and country. (2) Population characteristics: sample size, age, body mass index (BMI), cancer type, and surgical history. (3) Perioperative outcomes: hospital stay, total operative time, PALND operative time, estimated blood loss, and blood transfusion. (4) Surgical outcomes: aortic lymph nodes and conversion to laparotomy. (5) Intraoperative complications, postoperative overall complications (defined as Clavien grade ≥ 1), postoperative major complications (defined as Clavien grade ≥ 3) (14). Any dispute was resolved by consensus or consultation with a third reviewer.

### Assessment of Bias Risk

Among the studies, ROBINS-I was used to assess for bias risk in non-RCTs (15). (1) confounding, (2) selection of participants, (3) classification of exposures, (4) departures from intended exposures, (5) missing data, (6) measurement of outcomes, and (7) selection of the reported result. The quality of RCTs was evaluated according to the tool of Cochrane Collaboration (16). It includes random sequence generation, allocation concealment, blinding of participants, personnel and outcome assessment, incomplete outcome data, selective reporting, and other sources of bias.

### Statistical Analysis

Meta-analyses were performed using weighted mean differences (WMD) for continuous measures, while odds ratios (ORs) were used for the dichotomous outcomes. The results were reported with 95% CIs. Meta-analyses of continuous variables were pooled using the inverse variance method and dichotomous variables were performed using the Mantel–Haenszel method. Heterogeneity across the studies was assessed using the *I*^2^ statistic (17). Taking account of predictable substantial between-trial heterogeneity, a random-effect model was used to combine all summary data. Review Manager 5.4 software (Rvman 5.3, Copenhagen: the Nordic Cochrane Center, the Cochrane Collaboration, 2014) was used for results synthesis. *p* < 0.05 was considered as statistically significant.

### Subgroup Analysis

We performed a subgroup analysis based on the different minimally invasive surgery for PALND: laparoscopy and robotic-assisted.

### Sensitivity Analysis

The robustness of the estimates was assessed by sensitivity analyses according to sample size (excluding studies with <60 patients) and applied the leave-one-out method to exclude studies one at a time from the pooled effect. However, sensitivity analyses were not performed in comparing three or fewer studies.

### Publication Bias

The visual interpretation of funnel plot asymmetry is inherently subjective, so we also conducted the Egger test. On the contrary, when the test power was lacking when 10 or fewer studies were included, we could not evaluate the publication bias (18, 19).

## Results

### Study Characteristics

**Figure 1:**
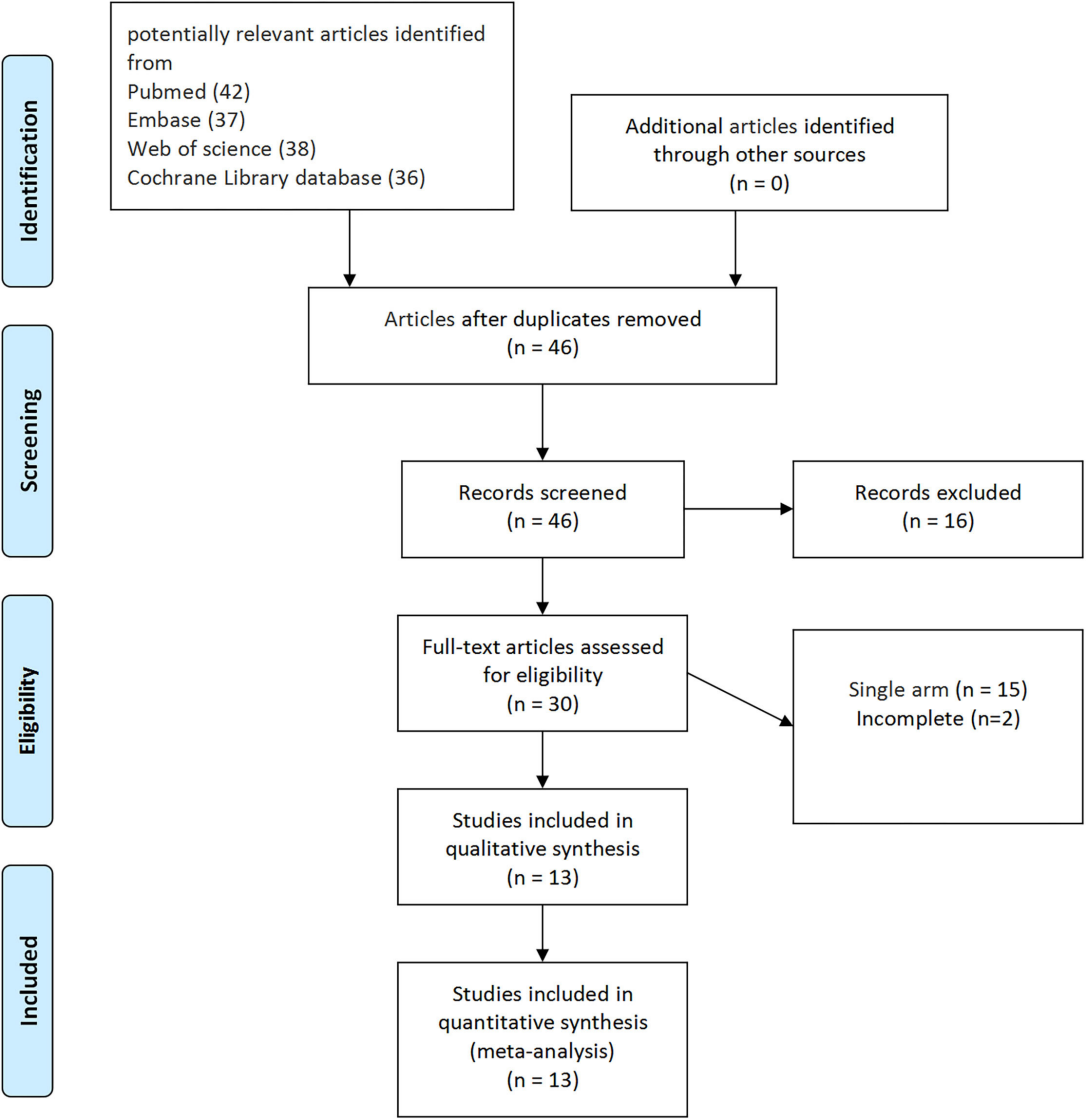
The Preferred Reporting Items for Systematic Reviews and Meta-Analyses (PRISMA) flow diagram for the systematic review.

According to the literature search and the inclusion criteria, we included 2,354 patients in 13 studies (3 RCTs and 10 non-RCTs) for meta-analysis (20–32). The 10 non-RCTs were all the retrospective comparisons (Figure 1). Table 1 summarizes the number and baseline characteristics of the included patients having their associated preoperative variables and each intervention (age, BMI, cancer type, and surgical history). The baseline characteristics of the BMI were not relatively equal in the three studies (the mean BMI was more in EP compared with TP in the three studies; *p* < 0.05) (24, 30, 31). However, the preoperative characteristics were comparable in other studies, with similar age, BMI, surgical history observed in each of the included studies. The patients in the two studies were divided into four groups, depending on their treatment type: TP laparoscopy, EP laparoscopy, TP robotic laparoscopy, and robot-assisted EP laparoscopy (20, 22).

**Table 1:** Baseline characteristics.

**Reference**	**Country**	**Age(y)**		**BMI (kg/m2)**		**Patients**		**Cancer type (n)**		**Surgical** **history (n)**		**Surgical approach**
		**EP**	**TP**	**EP**	**TP**	**EP**	**TP**	**EP**	**TP**	**EP**	**TP**	
Bebia et al. (20)	Spanish	63.6(11.3)	61.6(11.5)	28.9(6.7)	28.0(6.6)	68	62	Cervix 55 Ovary 13	Cervix 43 Ovary 19	38	36	Robotic
Bebia et al. (21)	Spanish	64.06(7.7)	65.4(9.85)	31.0(6.4)	30.8(6.6)	35	38	Cervix 32 Ovary 3	Cervix 36 Ovary 2	15	16	Laparoscopic
Díaz-Feijoo et al. (21)	Spain	63.7(10.2)	63.0(11.0)	29.6(6.6)	29.1(6.7)	103	100	Cervix 87 Ovary 16	Cervix 79 Ovary 21	31	33	Laparoscopic
Kerbage et al. (22)	France	<60:527 (n) ≥60:140 (n)	<60:145 (n) ≥60:49 (n)	<30:491 (n) ≥30:119 (n)	<30:161 (n) ≥30:22 (n)	681	198	Cervix 572 Endometrium 41 Ovary 54 Vagina 2	Cervix 104 Endometrium 41 Ovary 39	NA	NA	Robotic
Kerbage et al. (22)	France	<60:31 (n) ≥60:12 (n)	<60:87 (n) ≥60:44 (n)	<30:31 (n) ≥30:13 (n)	<30:115 (n) ≥30:12 (n)	44	135	Cervix 37 Endometrium 47	Cervix 78 Endometrium 35 Ovary 18	NA	NA	Laparoscopic
Salhi et al. (23)	France	52.9(12.5)	56.4(12.9)	25.2(5.5)	25.6(4.5)	69	74	Cervix 63 Endometrium 5 Ovary 1	Cervix 29 Endometrium 38 Ovary 7	40	31	Laparoscopic
Beytout et al. (24)	France	57(13.5)	56(14.5)	24.0(5.75)	23(5.5)	44	56	Cervix 27 Endometrium 14 Ovary 1 Vagina 2	Cervix 20 Endometrium 25 Ovary 11	NA	NA	Laparoscopic
Díaz-Feijoo et al. (25)	Spain	65(13)	65(12.75)	29.0(6.15)	26.4(5.06)	31	29	Cervix 26 Endometrium 5	Cervix 22 Endometrium 7	14	17	Laparoscopic
O'Hanlan et al. (26)	USA	58(11.5)	57(12.75)	26.0(5)	26(5.5)	79	36	Cervix 4 Endometrium 53 Ovary 20	Cervix 0 Endometrium 53 Ovary 20	NA	NA	Laparoscopic
Akladios et al. (27)	France	55.2(9.25)	53.3(14.75)	26.9(7.25)	23.5(4.75)	21	51	Cervix 16 Endometrium 3 Ovary 1	Cervix 22 Endometrium 20 Ovary 8	12	22	Laparoscopic
Naoura et al. (28)	France	56(14)	54(14)	26.0(6)	24.0(4)	67	62	Cervix 41 Endometrium 22 Ovary 1 Vagina 3	Cervix 22 Endometrium 29 Ovary 11	49	39	Laparoscopic
Pakish et al. (29)	USA	62(11)	61(17)	35.1(6.7)	28.4(9.08)	34	108	Endometrium 34	Endometrium 108	NA	NA	Laparoscopic
Morales et al. (30)	Spain	52.4(12.4)	60.5(12.9)	28.6(41)	25.1(3.9)	28	19	Cervix 19 Endometrium 6 Ovary 3	Cervix 9 Endometrium 7 Ovary 3	NA	NA	Laparoscopic
Lambaudie et al. (31)	France	49(11.3)	43.1(8.8)	27.4(5.16)	22(3.4)	15	24	Cervix 14 Ovary 1	Cervix 24	NA	NA	Laparoscopic
Fleming et al. (32)	USA	65(8.25)	61(8.5)	29.0(4.5)	29.0(6.5)	19	24	Endometrium 19	Endometrium 24	NA	NA	Laparoscopic

*BMI, body mass index; EP, extraperitoneal; TP, transperitoneal; Mean (SD)*.

Perioperative and surgical outcomes are summarized in Table 2. The complications are summarized in Table 3, which includes intraoperative complications, postoperative overall complications, and postoperative major complications.

**Table 2:** Perioperative and surgical outcomes.

**Reference**	**hospital**		**Total operative**		**PALND operative**		**Estimated blood**		**Blood**		**Aortic lymph**		**Conversion to**	
	**stay (day)**		**time (min)**		**time (min)**		**loss (ml)**		**transfusion (n)**		**nodes (n)**		**laparotomy (n)**	
	**EP**	**TP**	**EP**	**TP**	**EP**	**TP**	**EP**	**TP**	**EP**	**TP**	**EP**	**TP**	**EP**	**TP**
Bebia et al. (20)	3(0.5)	3(0.5)	282.5(25)	282.5(18.75)	90(11.5)	90(10.0)	150(56.25)	200(57.5)	NA	NA	14.5(2.5)	13(2.25)	8	5
Bebia et al. (21)	2(0.25)	2(0.5)	270(18.75)	261(15)	90(6.25)	90(11.88)	105(17.5)	100(32.5)	NA	NA	13(1.75)	10(3)	1	1
Díaz-Feijoo et al. (21)	3(1.48)	3(1.48)	275(70.37)	270(44.4)	90(35.6)	90(37.0)	150(107.41)	110(111.1)	NA	NA	14(6.67)	12(7.41)	9	6
Kerbage et al. (22)	3(10)	3(5.75)	207.1(73.4)	214.6(88.3)	NA	NA	NA	NA	NA	NA	18(13)	14(12.5)	NA	NA
Kerbage et al. (22)	3(3.75)	3(4.5)	220.2(63.9)	257.1(117.1)	NA	NA	115.8(134.5)	141.5(265.7)	NA	NA	20(11.75)	13(12.25)	NA	NA
Salhi et al. (23)	4.2(2.6)	5(2.3)	166(77.8)	220.8(74.6)	NA	NA	191.1(197.0)	130(172)	NA	NA	11.7(6.3)	12.9(7.2)	NA	NA
Beytout et al. (24)	2.93(1.6)	3.72(2)	NA	NA	94(44)	110(43)	NA	NA	NA	NA	12(6)	16(6.5)	NA	NA
Díaz-Feijoo et al. (25)	3(1.5)	3(15)	260(57.5)	255(60)	90(27.5)	90(28.75)	105(97.5)	100(248.75)	3	1	12(9.25)	13(6.25)	2	1
O'Hanlan et al. (26)	1.4(1.25)	1.1(1)	240(66.25)	202(56)	NA	NA	NA	NA	NA	NA	14(7)	10(9.5)	1	3
Akladios et al. (27)	5.6(3)	7.7(9.5)	NA	NA	125.7(33.75)	200.2(81.25)	NA	NA	1	9	13(5.5)	17(8.25)	NA	NA
Naoura et al. (28)	3.2(2)	3.7(2)	NA	NA	108(62)	110(41)	NA	NA	3	3	12(6)	15(5)	3	4
Pakish et al. (29)	NA	NA	NA	NA	NA	NA	NA	NA	1	7	10(4.5)	5(5.75)	NA	NA
Morales et al. (30)	4.6(3.18)	4.5(1.6)	173(51)	211(38)	NA	NA	NA	NA	NA	NA	15(5.9)	17.4(8.6)	NA	NA
Lambaudie et al. (31)	2.6(0.6)	3.1(1.6)	202.1(6.4)	163.7(6)	NA	NA	70.9(69.3)	154.6(171)	NA	NA	16.7(8)	13.3(7.3)	NA	NA
Fleming et al. (32)	1.4(1.25)	1(1)	190(30)	179(30)	NA	NA	100(993.76)	100(137.5)	NA	NA	9(6.75)	7(3.75)	NA	NA

*EP, extraperitoneal; TP, transperitoneal; Mean (SD)*.

**Table 3:** Complication.

**Reference**	**Intraoperative**		**Postoperative**		**Postoperative**	
			**(Clavien**		**(Clavien**	
			**grade** **≥1)**		**grade** **≥3)**	
	**EP**	**TP**	**EP**	**TP**	**EP**	**TP**
Bebia et al. (20)	2	6	17	15	2	1
Bebia et al. (21)	0	1	2	11	0	0
Díaz-Feijoo et al. (21)	2	7	3	1	1	1
Kerbage et al. (22)	NA	NA	86	23	78	15
Kerbage 2020 (b)	NA	NA	7	6	6	5
Salhi et al. (23)	4	9	12	9	NA	NA
Beytout et al. (24)	2	6	4	15	NA	NA
Díaz-Feijoo et al. (25)	0	4	7	12	5	9
O'Hanlan et al. (26)	2	4	NA	NA	NA	NA
Akladios et al. (27)	0	3	5	16	2	1
Naoura et al. (28)	3	6	7	15	NA	NA
Pakish et al. (29)	1	6	14	51	NA	NA
Morales et al. (30)	2	0	NA	NA	NA	NA
Lambaudie et al. (31)	NA	NA	1	1	NA	NA
Fleming et al. (32)	1	1	3	3	NA	NA

### Assessment of Quality

**Figure 2:**
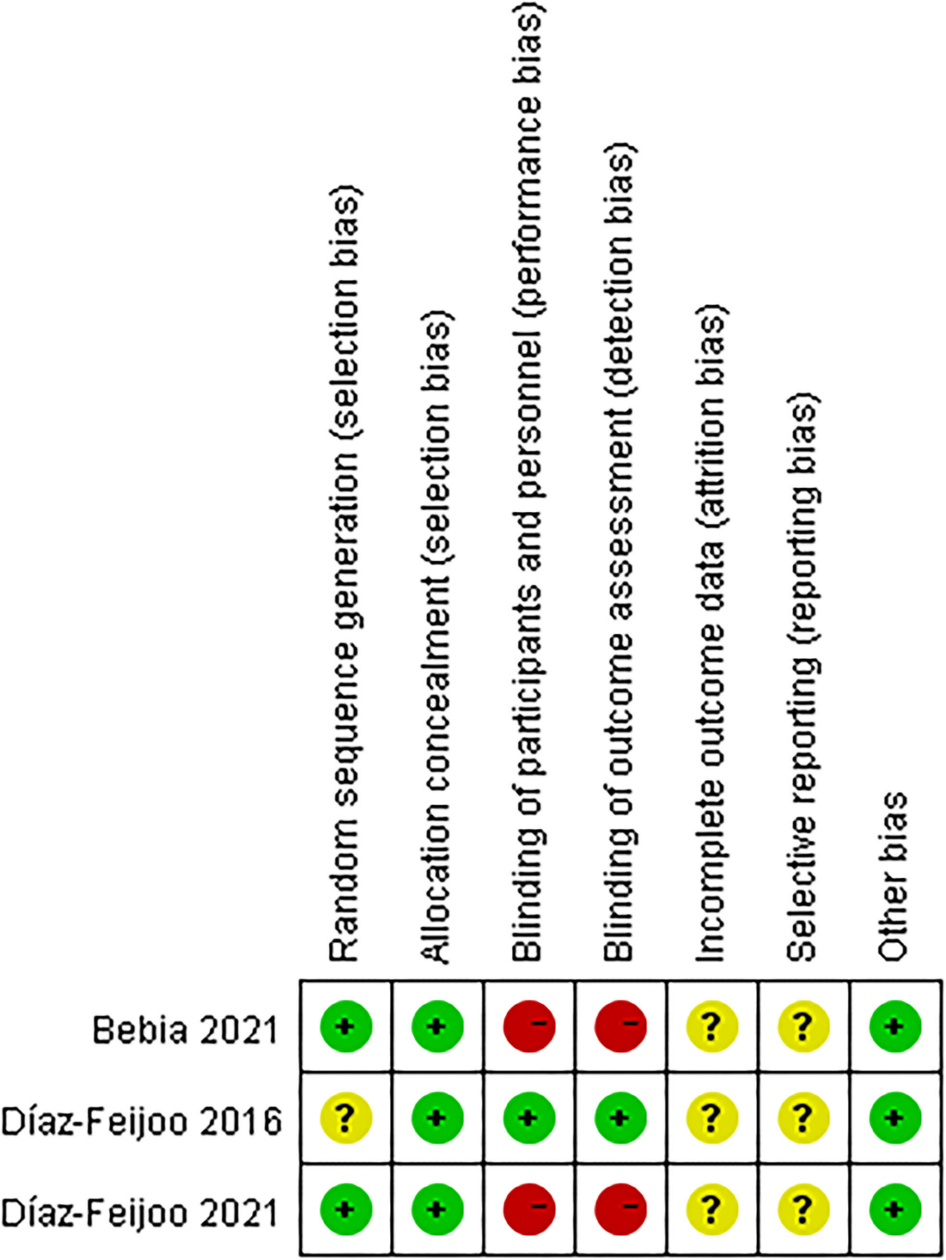
Risk of bias assessment [randomized control trials (RCTs)].

The three RCTs were not double-blinded, the level of evidence was low. So, the three RCT studies had an intermediate risk of bias (Figure 2). On the other hand, because the 10 non-RCTs conducted retrospective comparative analysis, the level of evidence was intermediate. Overall, the risk of bias was considered moderate in 10 studies (Table 4; the details are in the Supplementary Material).

**Table 4:** The risk of bias [nonrandomized controlled trials (RCTs)]-ROBINS-I.

**Bias domain**	**Kerbage et al. (22)**	**Salhi et al. (23)**	**Beytout et al. (24)**	**O'Hanlan et al. (26)**	**Akladios et al. (27)**	**Naoura et al. (28)**	**Pakish et al. (29)**	**Morales et al. (30)**	**Lambaudie et al. (31)**	**Fleming et al. (32)**
Bias due to confounding	Moderate	Moderate	Moderate	Moderate	Moderate	Moderate	Moderate	Moderate	Moderate	Moderate
Bias in selection of participants into the study	Low	Low	Low	Low	Low	Low	Low	Low	Low	Low
Bias in classification of interventions	Low	Low	Low	Moderate	Low	Moderate	Low	Moderate	Low	Low
Bias due to deviations from intended interventions	Low	Low	Moderate	Low	Low	Low	Low	Low	Low	Low
Bias due to missing data	Low	Low	Low	Low	Low	Low	Low	Low	Low	Low
Bias in measurement of outcomes	Low	Moderate	Moderate	Low	Moderate	Low	Moderate	Moderate	Low	Moderate
Bias in selection of the reported result	Moderate	Moderate	Moderate	Moderate	Moderate	Moderate	Moderate	Moderate	Moderate	Moderate
Overall bias	Moderate	Moderate	Moderate	Moderate	Moderate	Moderate	Moderate	Moderate	Moderate	Moderate

## Outcome Analysis

### Perioperative Outcomes

**Figure 3:**
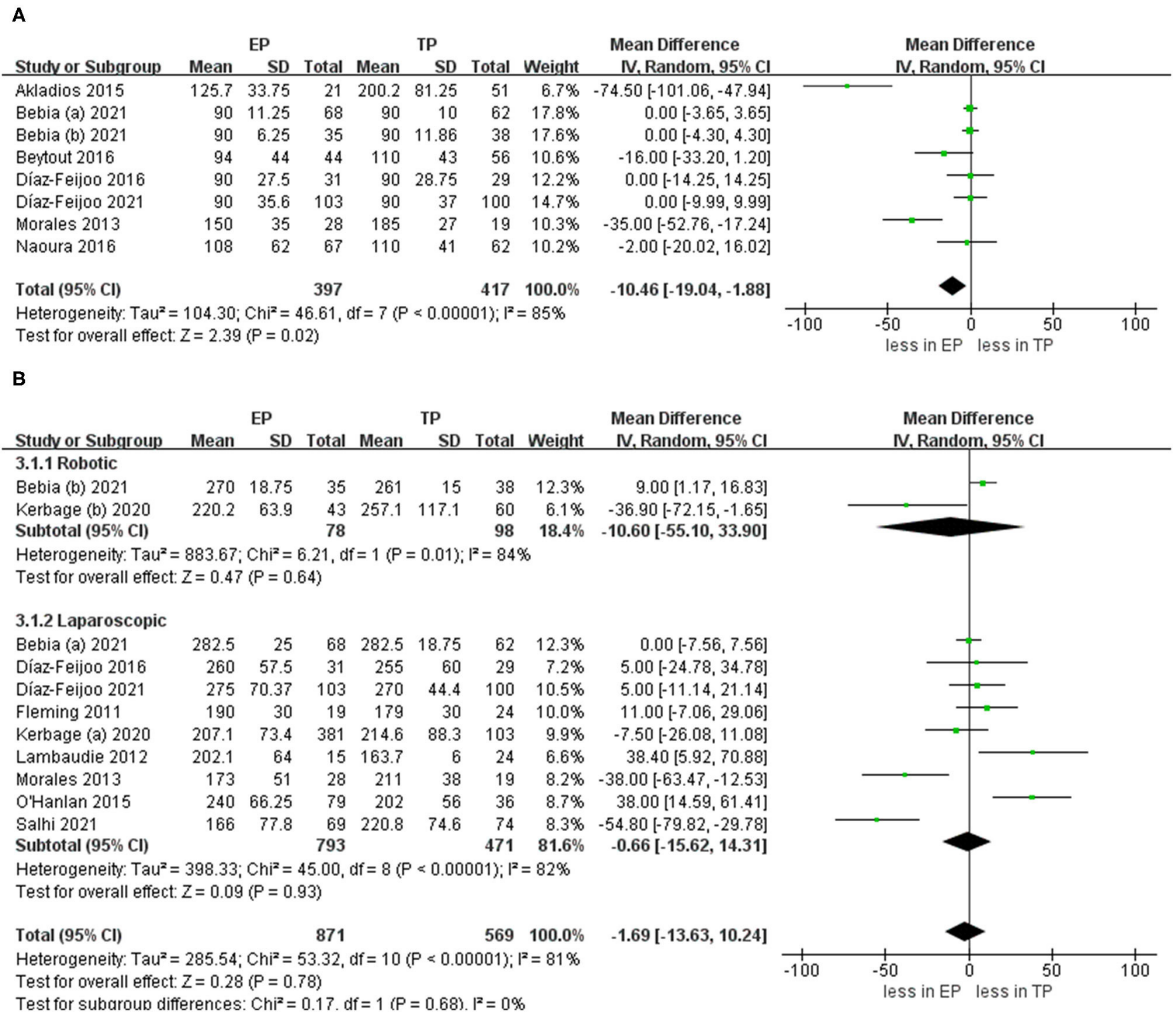
Forest plots of para-aortic lymphadenectomy (PALND) operative time and total operative time for extraperitoneal (EP) vs. transperitoneal (TP). **(A)** PALND operative time; **(B)** total operative time. IV, inverse variance; DF, degrees of freedom.

When we pooled the results from the seven studies, the EP group demonstrated less PALND operative time compared to TP (WMD −10.46 min, 95% CI −19.04, −1.88; *p* = 0.02) (20, 21, 24, 25, 27, 28, 30). However, the sensitivity analysis showed no significant differences with the removal of two studies (23, 26) (*p* = 0.16, *p* = 0.10). It shows that the estimates were not robust. Pooled analysis in nine studies showed no significant differences in the total operative time (*p* = 0.78) (6, 9, 21–24, 30–32). After we performed the leave-one-out test eliminated studies with <60 patients and performed the leave-one-out test, the statistical significance did not change in the terms of total operative time (Figure 3).

**Figure 4:**
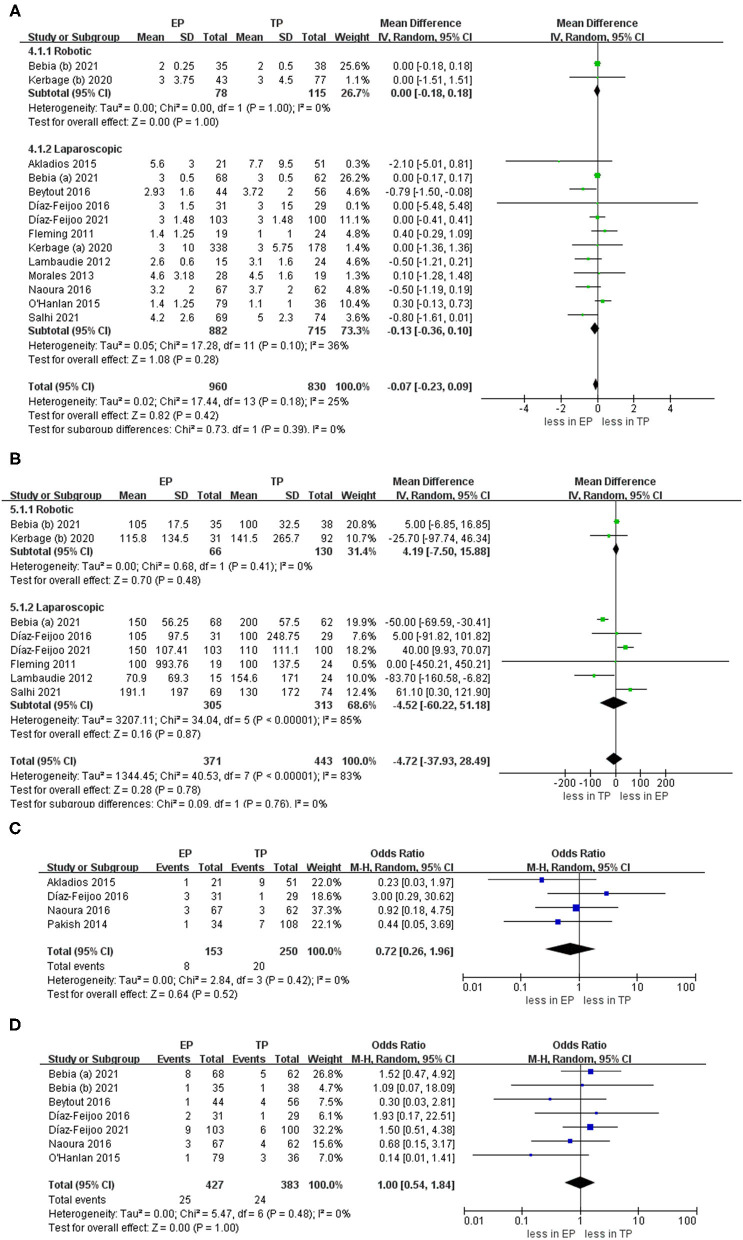
Forest plots of perioperative outcomes for EP vs. TP **(A)** hospital stay; **(B)** estimated blood loss; **(C)** blood transfusion; **(D)** conversion to laparotomy. IV, inverse variance; DF, degrees of freedom.

There is no statistical significance in the hospital stay between EP and TP (twelve studies (*p* = 0.42) (20–28, 30–32). Pooled analysis in six studies showed no significant differences in estimated blood loss (*p* = 0.78) (20–25, 31, 32). In addition, pooling results from four studies (25, 27–29) no significant differences were found in blood transfusion (*p* = 0.52). Furthermore, there was no statistical significance in the conversion to laparotomy between EP and TP (six studies; *p* = 1.00) (20, 21, 24–26, 28). Leave-one-out sensitivity analysis and excluding smaller studies also showed no significant differences between EP and TP in the terms of hospital stay, blood loss, blood transfusion, and conversion to laparotomy (Figure 4).

### Aortic Lymph Nodes

**Figure 5:**
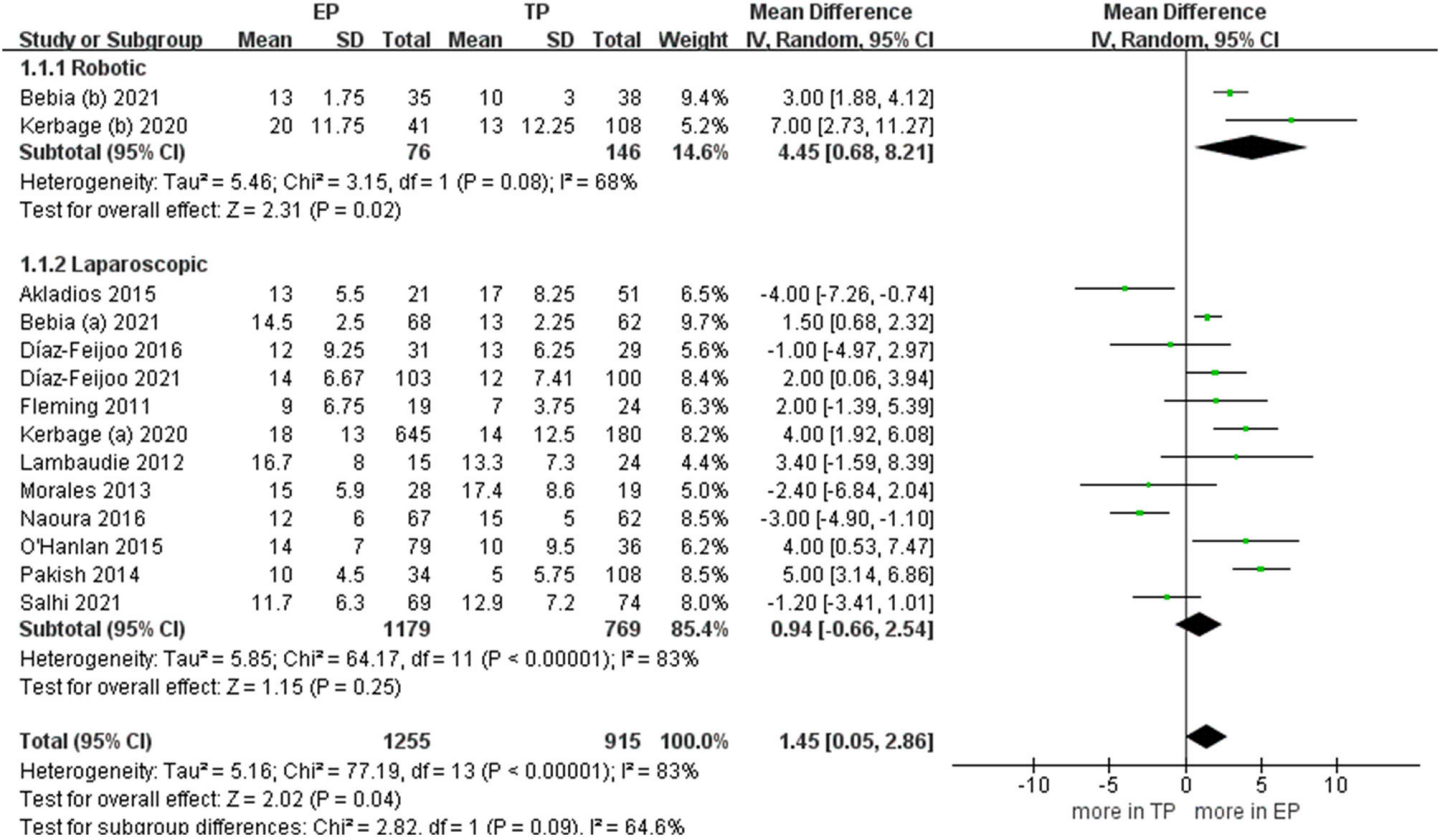
Forest plots of aortic lymph nodes for EP vs. TP. IV, inverse variance; DF, degrees of freedom.

During the aortic lymph nodes, fewer lymph nodes were removed in group TP compared with the group EP (WMD 1.45, 95% CI 0.05, 2.86; *p* = 0.04) (20–23, 25–32). However, no significant differences between the two groups were seen in the laparoscopic subgroup. Furthermore, after we eliminated studies with <60 patients and performed the leave-one-out test, the statistical significance changed in the terms of lymph nodes. Therefore, it proved that the estimates were not robust and the heterogeneity was high. We would discuss this further in the discussion section (Figure 5).

### Complications

**Figure 6:**
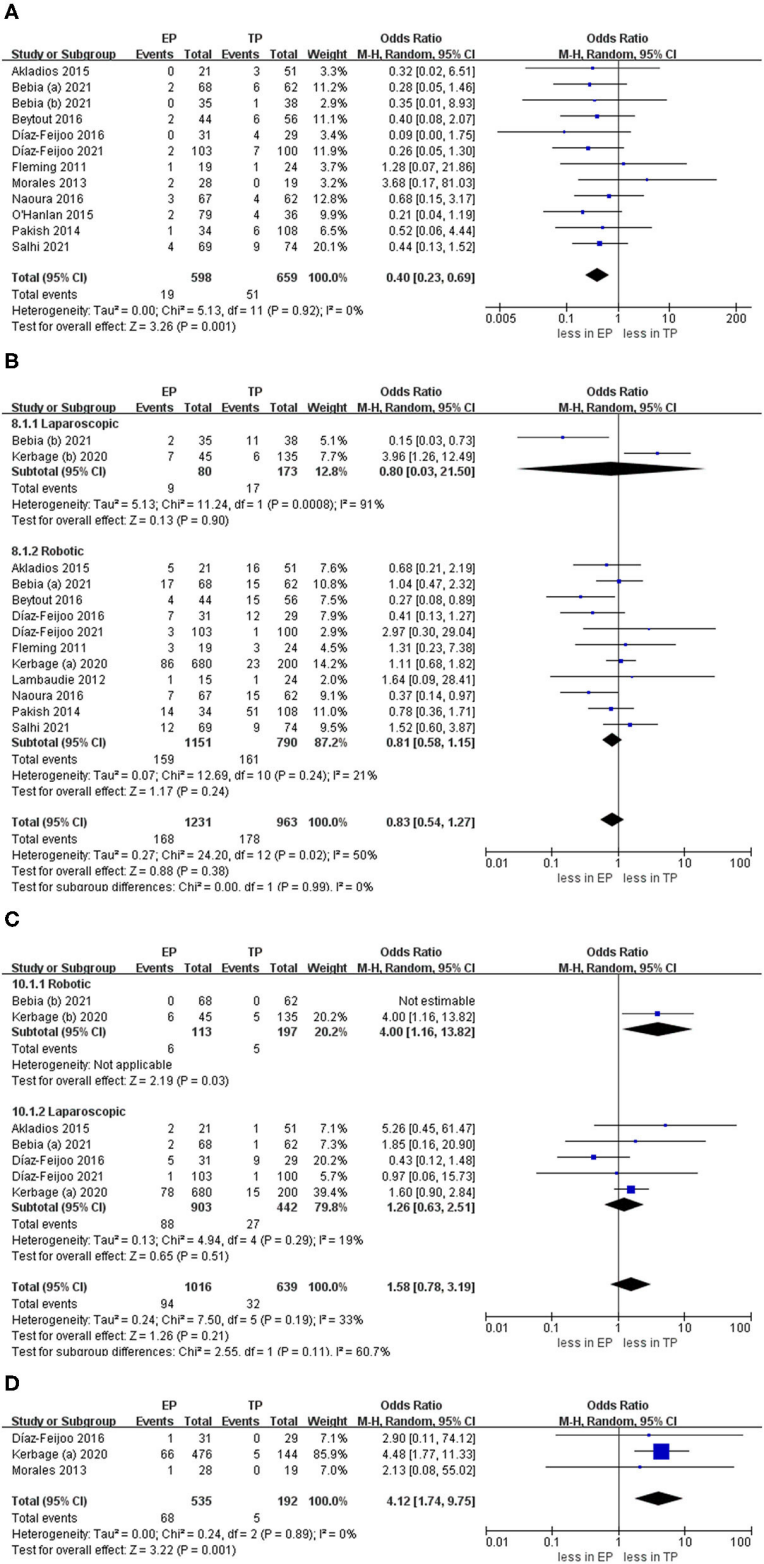
Forest plots of complication outcomes for EP vs. TP. **(A)** Intraoperative complication; **(B)** postoperative overall complications (Clavien grade ≥ 1); **(C)** postoperative major complications (Clavien grade ≥ 3); **(D)** lymphocele). IV, inverse variance; DF, degrees of freedom.

The intraoperative complication rates were 3.17 % (19 out of 598 cases) for EP and 7.74 % (51 of 659 cases) for TP, respectively (20, 21, 23–30, 32). The pooled estimates of intraoperative complication were less in EP compared with TP (eleven studies; OR 0.40, 95% CI 0.23, 0.69; *p* = 0.001). However, no significant differences were found in the postoperative overall complications (Clavien grade ≥1) and postoperative major complications (Clavien grade ≥3) (*p* = 0.24; *p* = 0.51). Furthermore, after we eliminated studies with <60 patients and performed the leave-one-out test, the statistical significance did not change in the terms of intraoperative, overall, and major complications. However, in terms of postoperative complications about lymphocele, TA was associated with fewer complications compared with EP (OR 4.12, 95% CI 1.74, 9.75; *p* = 0.001) (Figure 6).

### Heterogeneity

Most of the outcomes had moderate-to-low heterogeneity. High heterogeneity was found in the aortic lymph nodes, operative time, and blood loss. However, this is not surprised given the economic and cultural differences in the healthcare institutions worldwide publishing their data on EA and TA.

### Publication Bias Assessment

**Figure 7:**
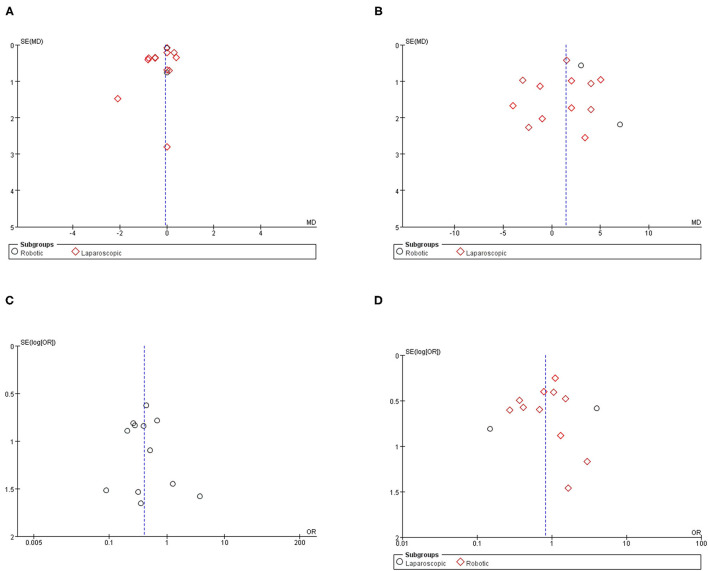
Funnel plot for the evaluation of potential publication bias. **(A)** Hospital stay; **(B)** aortic lymph nodes; **(C)** intraoperative complication; **(D)** postoperative overall complication.

Funnel plots are given in Figure 7. The results of Egger tests showed no evidence for the publication bias for hospital stay (*p* = 0.62), aortic lymph nodes (*p* = 0.70), overall complications (*p* = 0.78), and intraoperative complication (*p* = 0.54).

## Discussion

We report 11 comparative outcomes analyses of EP vs. TP. It includes 2,354 patients from 13 studies. Some conclusions drawn from the study are worthy of in-depth discussion.

### Perioperative Outcomes

In the EP group, PALND operative time was less compared with the TP group. From a technical point of view, the main advantage of the EP is the avoidance of intra-abdominal entry. It allowed for relatively rapid access to the vascular axes, especially the left aortic group of the lymph nodes until the level of the renal vein (33). Some studies also reported that the EP approach was associated with less PALND operative time over the TP approach (34, 35). However, after the removal of two studies, the sensitivity analysis showed no significant differences. On the other hand, because of the lack of study, we cannot perform a subgroup analysis based on the different surgical approaches for the PALND. Therefore, caution should be taken in evaluating the PALND operative time between EP and TA. In terms of total operative time, no significant differences were found between EP and TP. The outcomes of this study about operative time largely align with those of Prodromidou et al. (12).

Results showed that there was no statistical significance in the hospital stay between EP and TP. However, surgeon and institutional volume are consistently shown to be important factors impacting the outcome of minimally invasive surgeries and PALND is no exception: a reduction in hospital stay and operative time with operative volume was demonstrated by Greco et al. (36). On the other hand, the cumulative analysis showed no significant difference between EP and TP in terms of estimated blood loss and blood transfusion. The technical difficulties of the EP approach include conversion to laparotomy due to greater difficulty in resolving intraoperative lesions, or accidental rupture of the peritoneum. However, in our study, the conversion to laparotomy rates were 5.85 % (25 out of 427 cases) for EP and 6.27 % (24 of 383 cases) for TP, respectively. Furthermore, previous studies have shown that conversion to laparotomy rates is similar between EP and TP about 6.0–7.0% that are almost consistent with our study (27, 29). It is worth emphasizing that for patients with an ovarian malignancy or endometrial, conversion to laparotomy might be related to the need of performing other surgical procedures as part of the treatment of the disease, but not exclusively related to the procedure of PALND.

### Aortic Lymph Nodes

More lymph nodes were removed in group EP compared with the group TP. However, no significant differences were found in the laparoscopic subgroup. First, the explanation for this inconsistency between studies is that in a large number of robotic TP procedures in an included study. The double docking was not achieved. Therefore, it prevented a more extensive lymph node dissection (22). In other words, the lymph node dissection rate might be influenced by the fact that the robotic procedures were performed with the older generation (37). Second, the mean BMI was more in EP compared with TP in the three studies (*P* < 0.05). Due to increased intra-abdominal adipose tissue, as the BMI increases, TP lymphadenectomy may not be sufficient to collect the lymph nodes of the renal blood vessels because of the poor visualization (38). There is no doubt that the difficulty of lymphadenectomy would be lower in the TP group because of the BMI. The EP approach could better enter the left aortic lymph nodes, especially into the challenging supra mesenteric lymph node group. There was no intestinal disturbance during the nodule dissection, and the left ureter was always visible (39). Therefore, EP seems advantageous compared with TP in the lymphadenectomy. As mentioned earlier, in obese patients, a study reported that in a review of 206 patients with endometrial cancer presenting with a BMI > 35 kg/m^2^, the median number of paraaortic nodes removed in TP was less compared with EP (40). Furthermore, a study had also shown that this technology can be safely and systematically applied to obese patients. It can perform thorough sampling and inspection, and the number of retrieved lymph nodes was greater in obese patients operated on using the EP approach compared with those undergoing TP (41).

### Complications

The intraoperative complications were less in EP compared with TP. In terms of intraoperative complications, one advantage of the EP approach is the absence of bowel loops interposition in the surgical area and visualization of the ureters (42). Although, the finding was consistent with the previous meta-analysis results, the addition of six more well-powered studies comprising of 721 patients in this meta-analysis and the direct comparisons made in this study between EP and TP strengthen these findings. There is no statistical significance in the postoperative complications between EP and TP. However, TP was associated with less complications about lymphocele compared with EP, the first time that this has been demonstrated by meta-analysis. Postoperative lymphocele formation is the most reported complication associated with EP (30, 43). And marsupialization was a protective factor against lymphoceles. However, due to the insufficient outcomes and systematic marsupialization at the end of the EP surgery have not been evaluated prospectively. We could not arrive at a completely reliable conclusion. On the other hand, the postoperative complications might have been influenced by difficulties in distinguishing specific complications associated with PALND from the remaining complications associated with surgery. There, we have to be cautious when evaluating the postoperative complications after EP and TP.

To avoid heterogeneity and bias, we have performed a subgroup analysis was performed based on the different minimally invasive surgery. However, the subgroup analysis was not performed for all outcomes because the data were lacking. Previous reports on the robotic surgery have demonstrated that the operative time, the hospital stay, and the rate of complications were similar between the two approaches (11, 44). But the sample size of those studies is small, it is difficult for us to arrive at a reliable conclusion. Therefore, in the future, we would require more studies on different minimally invasive surgery to verify this conclusion. On the other hand, previous reports have demonstrated that the robotic assistance could lead to a reduction in the hospital stay, blood loss and an increase in lymph node retrieval count, and complications compared with conventional laparoscopy in the obese patients (45). Moreover, robotic-enhanced precision of movements and visualization could lead to a shorter time required to retrieve each lymph node (46), improving the efficiency of the operation. However, no specific criteria were used to decide whether the robotic assistance or laparoscopy was used to perform the procedures, we need more evidence to prove the superiority of robotic assistance and reveal the patients most likely to benefit from the robotic assistance. At last, the perioperative, surgical outcomes and complications in minimally invasive surgeries were also affected by the expertise and learning curves of the surgeon. The surgical outcomes could be improved after the initial learning curve was complete (39, 47). Furthermore, the robot-assisted PALND has a steep learning curve similar to that for laparoscopic surgery (48, 49). Thus, those factors we should be taken into account. So, more high-level evidence was required to prove those findings.

There are some limitations of this study. First, only three studies were RCTs vs. 10 non-RCTs that might have resulted in unmeasurable confounding factors and selection bias. Furthermore, some of the included studies had small sample sizes. There is no doubt that it is a limitation of our study. Second, the baseline characteristics between the two groups were not entirely comparable. Although, most studies showed no statistically significant difference in the age and surgical history between each patient group, the baseline characteristics of the BMI were not relatively equal in the three studies. Third, the included studies did not report individual results for each cancer. It represents a potential confounding factor in evaluating oncology results. Last, due to the lack of reported outcome data in the qualified studies, some of the results we aimed to analyze could not be included in the meta-analysis.

## Conclusion

There are no significant differences between EP and TP in terms of hospital stay, estimated blood loss, blood transfusion, conversion to laparotomy, total operative time, and postoperative complications. As expected, EP reported a lower rate of intraoperative complications as compared with TP, while more complications about lymphocele were found in the EP group. Furthermore, fewer lymph nodes were removed in group TP compared with the group EP. The main factors for choosing the approach are the habits of the patients, the location of the nodal disease, and importantly, the type of other procedures required to complete the surgery. Clinicians with advanced laparoscopic and/or robotics skills should be proficient in both techniques to suit the optimal approach of each patient. Furthermore, high-quality prospective randomized controlled trials should be conducted to compare the outcomes of different surgical approaches.

## Data Availability Statement

The original contributions presented in the study are included in the article/Supplementary Material, further inquiries can be directed to the corresponding author.

## Author Contributions

K-pL, X-zD, and TW worked on the protocol development, data collection and management, data analysis, and manuscript writing.

## Funding

This study was supported by the City of Nanchong Strategic Cooperation with Local Universities Foundation of Technology (20SXQT0305). This work was supported by the Application and Basic Research Program of the Sichuan Science and Technology Department (2020YJ0185).

## Conflict of Interest

The authors declare that the research was conducted in the absence of any commercial or financial relationships that could be construed as a potential conflict of interest.

## Publisher's Note

All claims expressed in this article are solely those of the authors and do not necessarily represent those of their affiliated organizations, or those of the publisher, the editors and the reviewers. Any product that may be evaluated in this article, or claim that may be made by its manufacturer, is not guaranteed or endorsed by the publisher.
