# Supplementary material for: Surgical Outcomes of Transperitoneal Para-Aortic Lymphadenectomy Compared With Extraperitoneal Approach in Gynecologic Cancers: A Systematic Review and Meta-Analysis
Source: Front Surg. 2021 Dec 21;8:779372. doi: 10.3389/fsurg.2021.779372 (PMC8724245; doi:10.3389/fsurg.2021.779372)
Supplement: Supplementary file 1 [file Data_Sheet_1.docx]

| The risk of bias In Non-randomized Studies – ROBINS-I | | |
| --- | --- | --- |
| **Kerbage 2020** |  |  |
| Bias domain | Signaling question | Response options |
| Bias due to confounding | 1.1 Is there potential for confounding of the  effect of intervention in this study? | Y |
|  |  |  |
|  | 1.2. Was the analysis based on splitting participants’ follow up time according to intervention received? | N |
|  |  |  |
|  | 1.3. Were intervention discontinuations or switches likely to be related to factors that are prognostic for the outcome? | NA |
|  |  |  |
|  | 1.4. Did the authors use an appropriate analysis method that  controlled for all the important confounding domains? | Y |
|  |  |  |
|  | 1.5. If Y/PY to 1.4: Were confounding domains that were controlled for measured validly and reliably by the variables available in this study? | Y |
|  |  |  |
|  |  |  |
|  | 1.6. Did the authors control for any post-intervention variables that could have been affected by the intervention? | NA |
|  |  |  |
|  | 1.7. Did the authors use an appropriate analysis method that  adjusted for all the important confounding domains and for time varying confounding? | NA |
|  |  |  |
|  |  |  |
|  | 1.8. If Y/PY to 1.7: Were confounding domains that were adjusted for measured validly and reliably by the variables available in this study? | NA |
|  |  |  |
|  |  |  |
|  | **Risk of bias judgement** | **Moderate** |
| Bias in selection of participants into the study | 2.1. Was selection of participants into the study (or into the analysis) based on participant characteristics observed after the start of intervention? | N |
|  |  |  |
|  |  |  |
|  | 2.2. If Y/PY to 2.1: Were the post intervention variables that influenced selection likely to be associated with intervention? | NA |
|  |  |  |
|  | 2.3 If Y/PY to 2.2: Were the post intervention variables that influenced selection likely to be influenced by the outcome or a cause of the outcome? | NA |
|  |  |  |
|  |  |  |
|  | 2.4. Do start of follow-up and start of  intervention coincide for most participants? | Y |
|  |  |  |
|  | 2.5. If Y/PY to 2.2 and 2.3, or N/PN to 2.4: Were adjustment techniques used that are likely to correct for the presence of selection biases? | NA |
|  |  |  |
|  |  |  |
|  | **Risk of bias judgement** | **Low** |
| Bias in classification of interventions | 3.1 Were intervention groups clearly defined? | Y |
|  |  |  |
|  | 3.2 Was the information used to define intervention groups recorded at the start of the intervention? | Y |
|  |  |  |
|  | 3.3 Could classification of intervention status have been affected by knowledge of the outcome or risk of the outcome? | N |
|  |  |  |
|  |  |  |
|  | **Risk of bias judgement** | **Low** |
| Bias due to deviations from intended interventions | 4.1. Were there deviations from the intended intervention beyond what would be expected in usual practice? | NA |
|  |  |  |
|  | 4.2. If Y/PY to 4.1: Were these deviations from intended intervention unbalanced between groups and likely to have affected the outcome? | NA |
|  |  |  |
|  |  |  |
|  | 4.3. Were important co-interventions balanced across intervention groups? | Y |
|  |  |  |
|  | 4.4. Was the intervention implemented successfully for most participants? | PY |
|  |  |  |
|  | 4.5. Did study participants adhere to the assigned intervention regimen? | Y |
|  |  |  |
|  | 4.6. If N/PN to 4.3, 4.4 or 4.5: Was an appropriate analysis used to estimate the effect of starting and adhering to the  intervention? | NA |
|  |  |  |
|  |  |  |
|  | **Risk of bias judgement** | **Low** |
| Bias due to missing data | 5.1 Were outcome data available for all, or nearly all, participants? | Y |
|  |  |  |
|  | 5.2 Were participants excluded due to missing data on intervention status? | N |
|  |  |  |
|  | 5.3 Were participants excluded due to missing data on other variables needed for the analysis? | Y |
|  |  |  |
|  | 5.4 If PN/N to 5.1, or Y/PY to 5.2 or 5.3: Are the proportion of participants and reasons for missing data similar across interventions? | Y |
|  |  |  |
|  |  |  |
|  | 5.5 If PN/N to 5.1, or Y/PY to 5.2 or 5.3: Is there evidence that results were robust to the presence of missing data? | NA |
|  |  |  |
|  |  |  |
|  | **Risk of bias judgement** | **Low** |
| Bias in measurement  of outcomes | 6.1 Could the outcome measure have been influenced by knowledge of the intervention received? | N |
|  |  |  |
|  | 6.2 Were outcome assessors aware of the intervention received by study participants? | Y |
|  |  |  |
|  | 6.3 Were the methods of outcome assessment comparable across intervention groups? | Y |
|  |  |  |
|  | 6.4 Were any systematic errors in measurement of the outcome related to intervention received? | N |
|  |  |  |
|  | **Risk of bias judgement** | **Low** |
| Bias in selection of the reported result | 7.1 multiple outcome measurements within the outcome domain? | N |
|  |  |  |
|  | 7.2 multiple analyses of the intervention outcome relationship? | N |
|  |  |  |
|  | 7.3 different subgroups? | N |
|  |  |  |
|  | **Risk of bias judgement** | **Moderate** |
|  | **Overall bias** | **Moderate** |
| Y: Yes; PY: Probably Yes; N: NO; NA: Not Applicable | | |
| **Salhi 2021** |  |  |
| Bias domain | Signaling question | Response options |
| Bias due to confounding | 1.1 Is there potential for confounding of the  effect of intervention in this study? | Y |
|  |  |  |
|  | 1.2. Was the analysis based on splitting participants’ follow up time according to intervention received? | N |
|  |  |  |
|  | 1.3. Were intervention discontinuations or switches likely to be related to factors that are prognostic for the outcome? | NA |
|  |  |  |
|  | 1.4. Did the authors use an appropriate analysis method that  controlled for all the important confounding domains? | Y |
|  |  |  |
|  | 1.5. If Y/PY to 1.4: Were confounding domains that were controlled for measured validly and reliably by the variables available in this study? | Y |
|  |  |  |
|  |  |  |
|  | 1.6. Did the authors control for any post-intervention variables that could have been affected by the intervention? | Y |
|  |  |  |
|  | 1.7. Did the authors use an appropriate analysis method that  adjusted for all the important confounding domains and for time varying confounding? | NA |
|  |  |  |
|  |  |  |
|  | 1.8. If Y/PY to 1.7: Were confounding domains that were adjusted for measured validly and reliably by the variables available in this study? | NA |
|  |  |  |
|  |  |  |
|  | **Risk of bias judgement** | **Moderate** |
| Bias in selection of participants into the study | 2.1. Was selection of participants into the study (or into the analysis) based on participant characteristics observed after the start of intervention? | N |
|  |  |  |
|  |  |  |
|  | 2.2. If Y/PY to 2.1: Were the post intervention variables that influenced selection likely to be associated with intervention? | NA |
|  |  |  |
|  | 2.3 If Y/PY to 2.2: Were the post intervention variables that influenced selection likely to be influenced by the outcome or a cause of the  outcome? | NA |
|  |  |  |
|  |  |  |
|  | 2.4. Do start of follow-up and start of  intervention coincide for most participants? | Y |
|  |  |  |
|  | 2.5. If Y/PY to 2.2 and 2.3, or N/PN to 2.4: Were adjustment techniques used that are likely to correct for the presence of selection biases? | NA |
|  |  |  |
|  |  |  |
|  | **Risk of bias judgement** | **Low** |
| Bias in classification of interventions | 3.1 Were intervention groups clearly defined? | Y |
|  |  |  |
|  | 3.2 Was the information used to define intervention groups recorded at the start of the intervention? | Y |
|  |  |  |
|  | 3.3 Could classification of intervention status have been affected by knowledge of the outcome or risk of the outcome? | N |
|  |  |  |
|  |  |  |
|  | **Risk of bias judgement** | **Low** |
| Bias due to deviations from intended interventions | 4.1. Were there deviations from the intended intervention beyond what would be expected in usual practice? | NA |
|  |  |  |
|  | 4.2. If Y/PY to 4.1: Were these deviations from intended intervention unbalanced between groups and likely to have affected the outcome? | NA |
|  |  |  |
|  |  |  |
|  | 4.3. Were important co-interventions balanced across intervention groups? | Y |
|  |  |  |
|  | 4.4. Was the intervention implemented successfully for most participants? | PY |
|  |  |  |
|  | 4.5. Did study participants adhere to the assigned intervention regimen? | PY |
|  |  |  |
|  | 4.6. If N/PN to 4.3, 4.4 or 4.5: Was an appropriate analysis used to estimate the effect of starting and adhering to the  intervention? | NA |
|  |  |  |
|  |  |  |
|  | **Risk of bias judgement** | **Low** |
| Bias due to missing data | 5.1 Were outcome data available for all, or nearly all, participants? | Y |
|  |  |  |
|  | 5.2 Were participants excluded due to missing data on intervention status? | N |
|  |  |  |
|  | 5.3 Were participants excluded due to missing data on other variables needed for the analysis? | N |
|  |  |  |
|  | 5.4 If PN/N to 5.1, or Y/PY to 5.2 or 5.3: Are the proportion of participants and reasons for missing data similar across interventions? | NA |
|  |  |  |
|  |  |  |
|  | 5.5 If PN/N to 5.1, or Y/PY to 5.2 or 5.3: Is there evidence that results were robust to the presence of missing data? | NA |
|  |  |  |
|  |  |  |
|  | **Risk of bias judgement** | **Low** |
| Bias in measurement  of outcomes | 6.1 Could the outcome measure have been influenced by knowledge of the intervention received? | N |
|  |  |  |
|  | 6.2 Were outcome assessors aware of the intervention received by study participants? | Y |
|  |  |  |
|  | 6.3 Were the methods of outcome assessment comparable across intervention groups? | Y |
|  |  |  |
|  | 6.4 Were any systematic errors in measurement of the outcome related to intervention received? | N |
|  |  |  |
|  | **Risk of bias judgement** | **Moderate** |
| Bias in selection of the reported result | 7.1 multiple outcome measurements within the outcome domain? | N |
|  |  |  |
|  | 7.2 multiple analyses of the intervention outcome relationship? | N |
|  |  |  |
|  | 7.3 different subgroups? | N |
|  |  |  |
|  | **Risk of bias judgement** | **Moderate** |
|  | **Overall bias** | **Moderate** |
| Y: Yes; PY: Probably Yes; N: NO; NA: Not Applicable | | |
| **Beytout 2016** |  |  |
| Bias domain | Signaling question | Response options |
| Bias due to confounding | 1.1 Is there potential for confounding of the  effect of intervention in this study? | Y |
|  |  |  |
|  | 1.2. Was the analysis based on splitting participants’ follow up time according to intervention received? | N |
|  |  |  |
|  | 1.3. Were intervention discontinuations or switches likely to be related to factors that are prognostic for the outcome? | NA |
|  |  |  |
|  | 1.4. Did the authors use an appropriate analysis method that  controlled for all the important confounding domains? | Y |
|  |  |  |
|  | 1.5. If Y/PY to 1.4: Were confounding domains that were controlled for measured validly and reliably by the variables available in this study? | Y |
|  |  |  |
|  |  |  |
|  | 1.6. Did the authors control for any post-intervention variables that could have been affected by the intervention? | Y |
|  |  |  |
|  | 1.7. Did the authors use an appropriate analysis method that  adjusted for all the important confounding domains and for time varying confounding? | NA |
|  |  |  |
|  |  |  |
|  | 1.8. If Y/PY to 1.7: Were confounding domains that were adjusted for measured validly and reliably by the variables available in this study? | NA |
|  |  |  |
|  |  |  |
|  | **Risk of bias judgement** | **Moderate** |
| Bias in selection of participants into the study | 2.1. Was selection of participants into the study (or into the analysis) based on participant characteristics observed after the start of intervention? | N |
|  |  |  |
|  |  |  |
|  | 2.2. If Y/PY to 2.1: Were the post intervention variables that influenced selection likely to be associated with intervention? | NA |
|  |  |  |
|  | 2.3 If Y/PY to 2.2: Were the post intervention variables that influenced selection likely to be influenced by the outcome or a cause of the  outcome? | NA |
|  |  |  |
|  |  |  |
|  | 2.4. Do start of follow-up and start of  intervention coincide for most participants? | Y |
|  |  |  |
|  | 2.5. If Y/PY to 2.2 and 2.3, or N/PN to 2.4: Were adjustment techniques used that are likely to correct for the presence of selection biases? | NA |
|  |  |  |
|  |  |  |
|  | **Risk of bias judgement** | **Low** |
| Bias in classification of interventions | 3.1 Were intervention groups clearly defined? | NY |
|  |  |  |
|  | 3.2 Was the information used to define intervention groups recorded at the start of the intervention? | Y |
|  |  |  |
|  | 3.3 Could classification of intervention status have been affected by knowledge of the outcome or risk of the outcome? | N |
|  |  |  |
|  |  |  |
|  | **Risk of bias judgement** | **Low** |
| Bias due to deviations from intended interventions | 4.1. Were there deviations from the intended intervention beyond what would be expected in usual practice? | NA |
|  |  |  |
|  | 4.2. If Y/PY to 4.1: Were these deviations from intended intervention unbalanced between groups and likely to have affected the outcome? | NA |
|  |  |  |
|  |  |  |
|  | 4.3. Were important co-interventions balanced across intervention groups? | Y |
|  |  |  |
|  | 4.4. Was the intervention implemented successfully for most participants? | Y |
|  |  |  |
|  | 4.5. Did study participants adhere to the assigned intervention regimen? | Y |
|  |  |  |
|  | 4.6. If N/PN to 4.3, 4.4 or 4.5: Was an appropriate analysis used to estimate the effect of starting and adhering to the  intervention? | NA |
|  |  |  |
|  |  |  |
|  | **Risk of bias judgement** | **Moderate** |
| Bias due to missing data | 5.1 Were outcome data available for all, or nearly all, participants? | Y |
|  |  |  |
|  | 5.2 Were participants excluded due to missing data on intervention status? | N |
|  |  |  |
|  | 5.3 Were participants excluded due to missing data on other variables needed for the analysis? | N |
|  |  |  |
|  | 5.4 If PN/N to 5.1, or Y/PY to 5.2 or 5.3: Are the proportion of participants and reasons for missing data similar across interventions? | NA |
|  |  |  |
|  |  |  |
|  | 5.5 If PN/N to 5.1, or Y/PY to 5.2 or 5.3: Is there evidence that results were robust to the presence of missing data? | NA |
|  |  |  |
|  |  |  |
|  | **Risk of bias judgement** | **Low** |
| Bias in measurement  of outcomes | 6.1 Could the outcome measure have been influenced by knowledge of the intervention received? | N |
|  |  |  |
|  | 6.2 Were outcome assessors aware of the intervention received by study participants? | Y |
|  |  |  |
|  | 6.3 Were the methods of outcome assessment comparable across intervention groups? | Y |
|  |  |  |
|  | 6.4 Were any systematic errors in measurement of the outcome related to intervention received? | N |
|  |  |  |
|  | **Risk of bias judgement** | **Moderate** |
| Bias in selection of the reported result | 7.1 multiple outcome measurements within the outcome domain? | N |
|  |  |  |
|  | 7.2 multiple analyses of the intervention outcome relationship? | N |
|  |  |  |
|  | 7.3 different subgroups? | N |
|  |  |  |
|  | **Risk of bias judgement** | **Moderate** |
|  | **Overall bias** | **Moderate** |
| Y: Yes; PY: Probably Yes; N: NO; NA: Not Applicable | | |
| **O'Hanlan 2015** |  |  |
| Bias domain | Signaling question | Response options |
| Bias due to confounding | 1.1 Is there potential for confounding of the  effect of intervention in this study? | PY |
|  |  |  |
|  | 1.2. Was the analysis based on splitting participants’ follow up time according to intervention received? | N |
|  |  |  |
|  | 1.3. Were intervention discontinuations or switches likely to be related to factors that are prognostic for the outcome? | NA |
|  |  |  |
|  | 1.4. Did the authors use an appropriate analysis method that  controlled for all the important confounding domains? | Y |
|  |  |  |
|  | 1.5. If Y/PY to 1.4: Were confounding domains that were controlled for measured validly and reliably by the variables available in this study? | Y |
|  |  |  |
|  |  |  |
|  | 1.6. Did the authors control for any post-intervention variables that could have been affected by the intervention? | Y |
|  |  |  |
|  | 1.7. Did the authors use an appropriate analysis method that  adjusted for all the important confounding domains and for time varying confounding? | NA |
|  |  |  |
|  |  |  |
|  | 1.8. If Y/PY to 1.7: Were confounding domains that were adjusted for measured validly and reliably by the variables available in this study? | NA |
|  |  |  |
|  |  |  |
|  | **Risk of bias judgement** | **Moderate** |
| Bias in selection of participants into the study | 2.1. Was selection of participants into the study (or into the analysis) based on participant characteristics observed after the start of intervention? | N |
|  |  |  |
|  |  |  |
|  | 2.2. If Y/PY to 2.1: Were the post intervention variables that influenced selection likely to be associated with intervention? | NA |
|  |  |  |
|  | 2.3 If Y/PY to 2.2: Were the post intervention variables that influenced selection likely to be influenced by the outcome or a cause of the  outcome? | NA |
|  |  |  |
|  |  |  |
|  | 2.4. Do start of follow-up and start of  intervention coincide for most participants? | Y |
|  |  |  |
|  | 2.5. If Y/PY to 2.2 and 2.3, or N/PN to 2.4: Were adjustment techniques used that are likely to correct for the presence of selection biases? | NA |
|  |  |  |
|  |  |  |
|  | **Risk of bias judgement** | **Low** |
| Bias in classification of interventions | 3.1 Were intervention groups clearly defined? | PY |
|  |  |  |
|  | 3.2 Was the information used to define intervention groups recorded at the start of the intervention? | Y |
|  |  |  |
|  | 3.3 Could classification of intervention status have been affected by knowledge of the outcome or risk of the outcome? | N |
|  |  |  |
|  |  |  |
|  | **Risk of bias judgement** | **Moderate** |
| Bias due to deviations from intended interventions | 4.1. Were there deviations from the intended intervention beyond what would be expected in usual practice? | NA |
|  |  |  |
|  | 4.2. If Y/PY to 4.1: Were these deviations from intended intervention unbalanced between groups and likely to have affected the outcome? | NA |
|  |  |  |
|  |  |  |
|  | 4.3. Were important co-interventions balanced across intervention groups? | Y |
|  |  |  |
|  | 4.4. Was the intervention implemented successfully for most participants? | Y |
|  |  |  |
|  | 4.5. Did study participants adhere to the assigned intervention regimen? | Y |
|  |  |  |
|  | 4.6. If N/PN to 4.3, 4.4 or 4.5: Was an appropriate analysis used to estimate the effect of starting and adhering to the  intervention? | NA |
|  |  |  |
|  |  |  |
|  | **Risk of bias judgement** | **Low** |
| Bias due to missing data | 5.1 Were outcome data available for all, or nearly all, participants? | Y |
|  |  |  |
|  | 5.2 Were participants excluded due to missing data on intervention status? | N |
|  |  |  |
|  | 5.3 Were participants excluded due to missing data on other variables needed for the analysis? | N |
|  |  |  |
|  | 5.4 If PN/N to 5.1, or Y/PY to 5.2 or 5.3: Are the proportion of participants and reasons for missing data similar across interventions? | NA |
|  |  |  |
|  |  |  |
|  | 5.5 If PN/N to 5.1, or Y/PY to 5.2 or 5.3: Is there evidence that results were robust to the presence of missing data? | NA |
|  |  |  |
|  |  |  |
|  | **Risk of bias judgement** | **Low** |
| Bias in measurement  of outcomes | 6.1 Could the outcome measure have been influenced by knowledge of the intervention received? | N |
|  |  |  |
|  | 6.2 Were outcome assessors aware of the intervention received by study participants? | Y |
|  |  |  |
|  | 6.3 Were the methods of outcome assessment comparable across intervention groups? | Y |
|  |  |  |
|  | 6.4 Were any systematic errors in measurement of the outcome related to intervention received? | N |
|  |  |  |
|  | **Risk of bias judgement** | **Low** |
| Bias in selection of the reported result | 7.1 multiple outcome measurements within the outcome domain? | N |
|  |  |  |
|  | 7.2 multiple analyses of the intervention outcome relationship? | N |
|  |  |  |
|  | 7.3 different subgroups? | N |
|  |  |  |
|  | **Risk of bias judgement** | **Moderate** |
|  | **Overall bias** | **Moderate** |
| Y: Yes; PY: Probably Yes; N: NO; NA: Not Applicable | | |
| **Akladios 2015** |  |  |
| Bias domain | Signaling question | Response options |
| Bias due to confounding | 1.1 Is there potential for confounding of the  effect of intervention in this study? | PY |
|  |  |  |
|  | 1.2. Was the analysis based on splitting participants’ follow up time according to intervention received? | N |
|  |  |  |
|  | 1.3. Were intervention discontinuations or switches likely to be related to factors that are prognostic for the outcome? | NA |
|  |  |  |
|  | 1.4. Did the authors use an appropriate analysis method that  controlled for all the important confounding domains? | Y |
|  |  |  |
|  | 1.5. If Y/PY to 1.4: Were confounding domains that were controlled for measured validly and reliably by the variables available in this study? | Y |
|  |  |  |
|  |  |  |
|  | 1.6. Did the authors control for any post-intervention variables that could have been affected by the intervention? | Y |
|  |  |  |
|  | 1.7. Did the authors use an appropriate analysis method that  adjusted for all the important confounding domains and for time varying confounding? | NA |
|  |  |  |
|  |  |  |
|  | 1.8. If Y/PY to 1.7: Were confounding domains that were adjusted for measured validly and reliably by the variables available in this study? | NA |
|  |  |  |
|  |  |  |
|  | **Risk of bias judgement** | **Moderate** |
| Bias in selection of participants into the study | 2.1. Was selection of participants into the study (or into the analysis) based on participant characteristics observed after the start of intervention? | N |
|  |  |  |
|  |  |  |
|  | 2.2. If Y/PY to 2.1: Were the post intervention variables that influenced selection likely to be associated with intervention? | NA |
|  |  |  |
|  | 2.3 If Y/PY to 2.2: Were the post intervention variables that influenced selection likely to be influenced by the outcome or a cause of the  outcome? | NA |
|  |  |  |
|  |  |  |
|  | 2.4. Do start of follow-up and start of  intervention coincide for most participants? | Y |
|  |  |  |
|  | 2.5. If Y/PY to 2.2 and 2.3, or N/PN to 2.4: Were adjustment techniques used that are likely to correct for the presence of selection biases? | NA |
|  |  |  |
|  |  |  |
|  | **Risk of bias judgement** | **Low** |
| Bias in classification of interventions | 3.1 Were intervention groups clearly defined? | NY |
|  |  |  |
|  | 3.2 Was the information used to define intervention groups recorded at the start of the intervention? | Y |
|  |  |  |
|  | 3.3 Could classification of intervention status have been affected by knowledge of the outcome or risk of the outcome? | N |
|  |  |  |
|  |  |  |
|  | **Risk of bias judgement** | **Low** |
| Bias due to deviations from intended interventions | 4.1. Were there deviations from the intended intervention beyond what would be expected in usual practice? | NA |
|  |  |  |
|  | 4.2. If Y/PY to 4.1: Were these deviations from intended intervention unbalanced between groups and likely to have affected the outcome? | NA |
|  |  |  |
|  |  |  |
|  | 4.3. Were important co-interventions balanced across intervention groups? | Y |
|  |  |  |
|  | 4.4. Was the intervention implemented successfully for most participants? | Y |
|  |  |  |
|  | 4.5. Did study participants adhere to the assigned intervention regimen? | Y |
|  |  |  |
|  | 4.6. If N/PN to 4.3, 4.4 or 4.5: Was an appropriate analysis used to estimate the effect of starting and adhering to the  intervention? | NA |
|  |  |  |
|  |  |  |
|  | **Risk of bias judgement** | **Low** |
| Bias due to missing data | 5.1 Were outcome data available for all, or nearly all, participants? | Y |
|  |  |  |
|  | 5.2 Were participants excluded due to missing data on intervention status? | N |
|  |  |  |
|  | 5.3 Were participants excluded due to missing data on other variables needed for the analysis? | N |
|  |  |  |
|  | 5.4 If PN/N to 5.1, or Y/PY to 5.2 or 5.3: Are the proportion of participants and reasons for missing data similar across interventions? | NA |
|  |  |  |
|  |  |  |
|  | 5.5 If PN/N to 5.1, or Y/PY to 5.2 or 5.3: Is there evidence that results were robust to the presence of missing data? | NA |
|  |  |  |
|  |  |  |
|  | **Risk of bias judgement** | **Low** |
| Bias in measurement  of outcomes | 6.1 Could the outcome measure have been influenced by knowledge of the intervention received? | N |
|  |  |  |
|  | 6.2 Were outcome assessors aware of the intervention received by study participants? | Y |
|  |  |  |
|  | 6.3 Were the methods of outcome assessment comparable across intervention groups? | Y |
|  |  |  |
|  | 6.4 Were any systematic errors in measurement of the outcome related to intervention received? | N |
|  |  |  |
|  | **Risk of bias judgement** | **Moderate** |
| Bias in selection of the reported result | 7.1 multiple outcome measurements within the outcome domain? | N |
|  |  |  |
|  | 7.2 multiple analyses of the intervention outcome relationship? | N |
|  |  |  |
|  | 7.3 different subgroups? | N |
|  |  |  |
|  | **Risk of bias judgement** | **Moderate** |
|  | **Overall bias** | **Moderate** |
| Y: Yes; PY: Probably Yes; N: NO; NA: Not Applicable | | |
| **Naoura 2016** |  |  |
| Bias domain | Signaling question | Response options |
| Bias due to confounding | 1.1 Is there potential for confounding of the  effect of intervention in this study? | PY |
|  |  |  |
|  | 1.2. Was the analysis based on splitting participants’ follow up time according to intervention received? | Y |
|  |  |  |
|  | 1.3. Were intervention discontinuations or switches likely to be related to factors that are prognostic for the outcome? | N |
|  |  |  |
|  | 1.4. Did the authors use an appropriate analysis method that  controlled for all the important confounding domains? | Y |
|  |  |  |
|  | 1.5. If Y/PY to 1.4: Were confounding domains that were controlled for measured validly and reliably by the variables available in this study? | Y |
|  |  |  |
|  |  |  |
|  | 1.6. Did the authors control for any post-intervention variables that could have been affected by the intervention? | Y |
|  |  |  |
|  | 1.7. Did the authors use an appropriate analysis method that  adjusted for all the important confounding domains and for time varying confounding? | NA |
|  |  |  |
|  |  |  |
|  | 1.8. If Y/PY to 1.7: Were confounding domains that were adjusted for measured validly and reliably by the variables available in this study? | NA |
|  |  |  |
|  |  |  |
|  | **Risk of bias judgement** | **Moderate** |
| Bias in selection of participants into the study | 2.1. Was selection of participants into the study (or into the analysis) based on participant characteristics observed after the start of intervention? | N |
|  |  |  |
|  |  |  |
|  | 2.2. If Y/PY to 2.1: Were the post intervention variables that influenced selection likely to be associated with intervention? | NA |
|  |  |  |
|  | 2.3 If Y/PY to 2.2: Were the post intervention variables that influenced selection likely to be influenced by the outcome or a cause of the  outcome? | NA |
|  |  |  |
|  |  |  |
|  | 2.4. Do start of follow-up and start of  intervention coincide for most participants? | Y |
|  |  |  |
|  | 2.5. If Y/PY to 2.2 and 2.3, or N/PN to 2.4: Were adjustment techniques used that are likely to correct for the presence of selection biases? | NA |
|  |  |  |
|  |  |  |
|  | **Risk of bias judgement** | **Low** |
| Bias in classification of interventions | 3.1 Were intervention groups clearly defined? | Y |
|  |  |  |
|  | 3.2 Was the information used to define intervention groups recorded at the start of the intervention? | PY |
|  |  |  |
|  | 3.3 Could classification of intervention status have been affected by knowledge of the outcome or risk of the outcome? | N |
|  |  |  |
|  |  |  |
|  | **Risk of bias judgement** | **Moderate** |
| Bias due to deviations from intended interventions | 4.1. Were there deviations from the intended intervention beyond what would be expected in usual practice? | NA |
|  |  |  |
|  | 4.2. If Y/PY to 4.1: Were these deviations from intended intervention unbalanced between groups and likely to have affected the outcome? | NA |
|  |  |  |
|  |  |  |
|  | 4.3. Were important co-interventions balanced across intervention groups? | Y |
|  |  |  |
|  | 4.4. Was the intervention implemented successfully for most participants? | Y |
|  |  |  |
|  | 4.5. Did study participants adhere to the assigned intervention regimen? | Y |
|  |  |  |
|  | 4.6. If N/PN to 4.3, 4.4 or 4.5: Was an appropriate analysis used to estimate the effect of starting and adhering to the  intervention? | NA |
|  |  |  |
|  |  |  |
|  | **Risk of bias judgement** | **Low** |
| Bias due to missing data | 5.1 Were outcome data available for all, or nearly all, participants? | Y |
|  |  |  |
|  | 5.2 Were participants excluded due to missing data on intervention status? | N |
|  |  |  |
|  | 5.3 Were participants excluded due to missing data on other variables needed for the analysis? | N |
|  |  |  |
|  | 5.4 If PN/N to 5.1, or Y/PY to 5.2 or 5.3: Are the proportion of participants and reasons for missing data similar across interventions? | NA |
|  |  |  |
|  |  |  |
|  | 5.5 If PN/N to 5.1, or Y/PY to 5.2 or 5.3: Is there evidence that results were robust to the presence of missing data? | NA |
|  |  |  |
|  |  |  |
|  | **Risk of bias judgement** | **Low** |
| Bias in measurement  of outcomes | 6.1 Could the outcome measure have been influenced by knowledge of the intervention received? | N |
|  |  |  |
|  | 6.2 Were outcome assessors aware of the intervention received by study participants? | Y |
|  |  |  |
|  | 6.3 Were the methods of outcome assessment comparable across intervention groups? | Y |
|  |  |  |
|  | 6.4 Were any systematic errors in measurement of the outcome related to intervention received? | N |
|  |  |  |
|  | **Risk of bias judgement** | **Low** |
| Bias in selection of the reported result | 7.1 multiple outcome measurements within the outcome domain? | N |
|  |  |  |
|  | 7.2 multiple analyses of the intervention outcome relationship? | N |
|  |  |  |
|  | 7.3 different subgroups? | N |
|  |  |  |
|  | **Risk of bias judgement** | **Moderate** |
|  | **Overall bias** | **Moderate** |
| Y: Yes; PY: Probably Yes; N: NO; NA: Not Applicable | | |
| **Pakish 2014** |  |  |
| Bias domain | Signaling question | Response options |
| Bias due to confounding | 1.1 Is there potential for confounding of the  effect of intervention in this study? | PY |
|  |  |  |
|  | 1.2. Was the analysis based on splitting participants’ follow up time according to intervention received? | N |
|  |  |  |
|  | 1.3. Were intervention discontinuations or switches likely to be related to factors that are prognostic for the outcome? | NA |
|  |  |  |
|  | 1.4. Did the authors use an appropriate analysis method that  controlled for all the important confounding domains? | Y |
|  |  |  |
|  | 1.5. If Y/PY to 1.4: Were confounding domains that were controlled for measured validly and reliably by the variables available in this study? | Y |
|  |  |  |
|  |  |  |
|  | 1.6. Did the authors control for any post-intervention variables that could have been affected by the intervention? | Y |
|  |  |  |
|  | 1.7. Did the authors use an appropriate analysis method that  adjusted for all the important confounding domains and for time varying confounding? | NA |
|  |  |  |
|  |  |  |
|  | 1.8. If Y/PY to 1.7: Were confounding domains that were adjusted for measured validly and reliably by the variables available in this study? | NA |
|  |  |  |
|  |  |  |
|  | **Risk of bias judgement** | **Moderate** |
| Bias in selection of participants into the study | 2.1. Was selection of participants into the study (or into the analysis) based on participant characteristics observed after the start of intervention? | N |
|  |  |  |
|  |  |  |
|  | 2.2. If Y/PY to 2.1: Were the post intervention variables that influenced selection likely to be associated with intervention? | NA |
|  |  |  |
|  | 2.3 If Y/PY to 2.2: Were the post intervention variables that influenced selection likely to be influenced by the outcome or a cause of the  outcome? | NA |
|  |  |  |
|  |  |  |
|  | 2.4. Do start of follow-up and start of  intervention coincide for most participants? | PY |
|  |  |  |
|  | 2.5. If Y/PY to 2.2 and 2.3, or N/PN to 2.4: Were adjustment techniques used that are likely to correct for the presence of selection biases? | NA |
|  |  |  |
|  |  |  |
|  | **Risk of bias judgement** | **Low** |
| Bias in classification of interventions | 3.1 Were intervention groups clearly defined? | Y |
|  |  |  |
|  | 3.2 Was the information used to define intervention groups recorded at the start of the intervention? | Y |
|  |  |  |
|  | 3.3 Could classification of intervention status have been affected by knowledge of the outcome or risk of the outcome? | N |
|  |  |  |
|  |  |  |
|  | **Risk of bias judgement** | **Low** |
| Bias due to deviations from intended interventions | 4.1. Were there deviations from the intended intervention beyond what would be expected in usual practice? | NA |
|  |  |  |
|  | 4.2. If Y/PY to 4.1: Were these deviations from intended intervention unbalanced between groups and likely to have affected the outcome? | NA |
|  |  |  |
|  |  |  |
|  | 4.3. Were important co-interventions balanced across intervention groups? | Y |
|  |  |  |
|  | 4.4. Was the intervention implemented successfully for most participants? | Y |
|  |  |  |
|  | 4.5. Did study participants adhere to the assigned intervention regimen? | Y |
|  |  |  |
|  | 4.6. If N/PN to 4.3, 4.4 or 4.5: Was an appropriate analysis used to estimate the effect of starting and adhering to the  intervention? | NA |
|  |  |  |
|  |  |  |
|  | **Risk of bias judgement** | **Low** |
| Bias due to missing data | 5.1 Were outcome data available for all, or nearly all, participants? | Y |
|  |  |  |
|  | 5.2 Were participants excluded due to missing data on intervention status? | N |
|  |  |  |
|  | 5.3 Were participants excluded due to missing data on other variables needed for the analysis? | N |
|  |  |  |
|  | 5.4 If PN/N to 5.1, or Y/PY to 5.2 or 5.3: Are the proportion of participants and reasons for missing data similar across interventions? | NA |
|  |  |  |
|  |  |  |
|  | 5.5 If PN/N to 5.1, or Y/PY to 5.2 or 5.3: Is there evidence that results were robust to the presence of missing data? | NA |
|  |  |  |
|  |  |  |
|  | **Risk of bias judgement** | **Low** |
| Bias in measurement  of outcomes | 6.1 Could the outcome measure have been influenced by knowledge of the intervention received? | N |
|  |  |  |
|  | 6.2 Were outcome assessors aware of the intervention received by study participants? | Y |
|  |  |  |
|  | 6.3 Were the methods of outcome assessment comparable across intervention groups? | PY |
|  |  |  |
|  | 6.4 Were any systematic errors in measurement of the outcome related to intervention received? | N |
|  |  |  |
|  | **Risk of bias judgement** | **Moderate** |
| Bias in selection of the reported result | 7.1 multiple outcome measurements within the outcome domain? | N |
|  |  |  |
|  | 7.2 multiple analyses of the intervention outcome relationship? | N |
|  |  |  |
|  | 7.3 different subgroups? | N |
|  |  |  |
|  | **Risk of bias judgement** | **Moderate** |
|  | **Overall bias** | **Moderate** |
| Y: Yes; PY: Probably Yes; N: NO; NA: Not Applicable | | |
| **Morales 2013** |  |  |
| Bias domain | Signaling question | Response options |
| Bias due to confounding | 1.1 Is there potential for confounding of the  effect of intervention in this study? | PY |
|  |  |  |
|  | 1.2. Was the analysis based on splitting participants’ follow up time according to intervention received? | N |
|  |  |  |
|  | 1.3. Were intervention discontinuations or switches likely to be related to factors that are prognostic for the outcome? | NA |
|  |  |  |
|  | 1.4. Did the authors use an appropriate analysis method that  controlled for all the important confounding domains? | Y |
|  |  |  |
|  | 1.5. If Y/PY to 1.4: Were confounding domains that were controlled for measured validly and reliably by the variables available in this study? | Y |
|  |  |  |
|  |  |  |
|  | 1.6. Did the authors control for any post-intervention variables that could have been affected by the intervention? | Y |
|  |  |  |
|  | 1.7. Did the authors use an appropriate analysis method that  adjusted for all the important confounding domains and for time varying confounding? | NA |
|  |  |  |
|  |  |  |
|  | 1.8. If Y/PY to 1.7: Were confounding domains that were adjusted for measured validly and reliably by the variables available in this study? | NA |
|  |  |  |
|  |  |  |
|  | **Risk of bias judgement** | **Moderate** |
| Bias in selection of participants into the study | 2.1. Was selection of participants into the study (or into the analysis) based on participant characteristics observed after the start of intervention? | N |
|  |  |  |
|  |  |  |
|  | 2.2. If Y/PY to 2.1: Were the post intervention variables that influenced selection likely to be associated with intervention? | NA |
|  |  |  |
|  | 2.3 If Y/PY to 2.2: Were the post intervention variables that influenced selection likely to be influenced by the outcome or a cause of the  outcome? | NA |
|  |  |  |
|  |  |  |
|  | 2.4. Do start of follow-up and start of  intervention coincide for most participants? | PY |
|  |  |  |
|  | 2.5. If Y/PY to 2.2 and 2.3, or N/PN to 2.4: Were adjustment techniques used that are likely to correct for the presence of selection biases? | NA |
|  |  |  |
|  |  |  |
|  | **Risk of bias judgement** | **Low** |
| Bias in classification of interventions | 3.1 Were intervention groups clearly defined? | Y |
|  |  |  |
|  | 3.2 Was the information used to define intervention groups recorded at the start of the intervention? | PY |
|  |  |  |
|  | 3.3 Could classification of intervention status have been affected by knowledge of the outcome or risk of the outcome? | N |
|  |  |  |
|  |  |  |
|  | **Risk of bias judgement** | **Moderate** |
| Bias due to deviations from intended interventions | 4.1. Were there deviations from the intended intervention beyond what would be expected in usual practice? | NA |
|  |  |  |
|  | 4.2. If Y/PY to 4.1: Were these deviations from intended intervention unbalanced between groups and likely to have affected the outcome? | NA |
|  |  |  |
|  |  |  |
|  | 4.3. Were important co-interventions balanced across intervention groups? | Y |
|  |  |  |
|  | 4.4. Was the intervention implemented successfully for most participants? | Y |
|  |  |  |
|  | 4.5. Did study participants adhere to the assigned intervention regimen? | Y |
|  |  |  |
|  | 4.6. If N/PN to 4.3, 4.4 or 4.5: Was an appropriate analysis used to estimate the effect of starting and adhering to the  intervention? | NA |
|  |  |  |
|  |  |  |
|  | **Risk of bias judgement** | **Low** |
| Bias due to missing data | 5.1 Were outcome data available for all, or nearly all, participants? | Y |
|  |  |  |
|  | 5.2 Were participants excluded due to missing data on intervention status? | N |
|  |  |  |
|  | 5.3 Were participants excluded due to missing data on other variables needed for the analysis? | N |
|  |  |  |
|  | 5.4 If PN/N to 5.1, or Y/PY to 5.2 or 5.3: Are the proportion of participants and reasons for missing data similar across interventions? | NA |
|  |  |  |
|  |  |  |
|  | 5.5 If PN/N to 5.1, or Y/PY to 5.2 or 5.3: Is there evidence that results were robust to the presence of missing data? | NA |
|  |  |  |
|  |  |  |
|  | **Risk of bias judgement** | **Low** |
| Bias in measurement  of outcomes | 6.1 Could the outcome measure have been influenced by knowledge of the intervention received? | N |
|  |  |  |
|  | 6.2 Were outcome assessors aware of the intervention received by study participants? | Y |
|  |  |  |
|  | 6.3 Were the methods of outcome assessment comparable across intervention groups? | PY |
|  |  |  |
|  | 6.4 Were any systematic errors in measurement of the outcome related to intervention received? | N |
|  |  |  |
|  | **Risk of bias judgement** | **Moderate** |
| Bias in selection of the reported result | 7.1 multiple outcome measurements within the outcome domain? | N |
|  |  |  |
|  | 7.2 multiple analyses of the intervention outcome relationship? | N |
|  |  |  |
|  | 7.3 different subgroups? | N |
|  |  |  |
|  | **Risk of bias judgement** | **Moderate** |
|  | **Overall bias** | **Moderate** |
| Y: Yes; PY: Probably Yes; N: NO; NA: Not Applicable | | |
| **Lambaudie 2012** |  |  |
| Bias domain | Signaling question | Response options |
| Bias due to confounding | 1.1 Is there potential for confounding of the  effect of intervention in this study? | PY |
|  |  |  |
|  | 1.2. Was the analysis based on splitting participants’ follow up time according to intervention received? | N |
|  |  |  |
|  | 1.3. Were intervention discontinuations or switches likely to be related to factors that are prognostic for the outcome? | NA |
|  |  |  |
|  | 1.4. Did the authors use an appropriate analysis method that  controlled for all the important confounding domains? | Y |
|  |  |  |
|  | 1.5. If Y/PY to 1.4: Were confounding domains that were controlled for measured validly and reliably by the variables available in this study? | Y |
|  |  |  |
|  |  |  |
|  | 1.6. Did the authors control for any post-intervention variables that could have been affected by the intervention? | Y |
|  |  |  |
|  | 1.7. Did the authors use an appropriate analysis method that  adjusted for all the important confounding domains and for time varying confounding? | NA |
|  |  |  |
|  |  |  |
|  | 1.8. If Y/PY to 1.7: Were confounding domains that were adjusted for measured validly and reliably by the variables available in this study? | NA |
|  |  |  |
|  |  |  |
|  | **Risk of bias judgement** | **Moderate** |
| Bias in selection of participants into the study | 2.1. Was selection of participants into the study (or into the analysis) based on participant characteristics observed after the start of intervention? | N |
|  |  |  |
|  |  |  |
|  | 2.2. If Y/PY to 2.1: Were the post intervention variables that influenced selection likely to be associated with intervention? | NA |
|  |  |  |
|  | 2.3 If Y/PY to 2.2: Were the post intervention variables that influenced selection likely to be influenced by the outcome or a cause of the  outcome? | NA |
|  |  |  |
|  |  |  |
|  | 2.4. Do start of follow-up and start of  intervention coincide for most participants? | PY |
|  |  |  |
|  | 2.5. If Y/PY to 2.2 and 2.3, or N/PN to 2.4: Were adjustment techniques used that are likely to correct for the presence of selection biases? | NA |
|  |  |  |
|  |  |  |
|  | **Risk of bias judgement** | **Low** |
| Bias in classification of interventions | 3.1 Were intervention groups clearly defined? | Y |
|  |  |  |
|  | 3.2 Was the information used to define intervention groups recorded at the start of the intervention? | Y |
|  |  |  |
|  | 3.3 Could classification of intervention status have been affected by knowledge of the outcome or risk of the outcome? | N |
|  |  |  |
|  |  |  |
|  | **Risk of bias judgement** | **Low** |
| Bias due to deviations from intended interventions | 4.1. Were there deviations from the intended intervention beyond what would be expected in usual practice? | NA |
|  |  |  |
|  | 4.2. If Y/PY to 4.1: Were these deviations from intended intervention unbalanced between groups and likely to have affected the outcome? | NA |
|  |  |  |
|  |  |  |
|  | 4.3. Were important co-interventions balanced across intervention groups? | Y |
|  |  |  |
|  | 4.4. Was the intervention implemented successfully for most participants? | Y |
|  |  |  |
|  | 4.5. Did study participants adhere to the assigned intervention regimen? | Y |
|  |  |  |
|  | 4.6. If N/PN to 4.3, 4.4 or 4.5: Was an appropriate analysis used to estimate the effect of starting and adhering to the  intervention? | NA |
|  |  |  |
|  |  |  |
|  | **Risk of bias judgement** | **Low** |
| Bias due to missing data | 5.1 Were outcome data available for all, or nearly all, participants? | Y |
|  |  |  |
|  | 5.2 Were participants excluded due to missing data on intervention status? | N |
|  |  |  |
|  | 5.3 Were participants excluded due to missing data on other variables needed for the analysis? | N |
|  |  |  |
|  | 5.4 If PN/N to 5.1, or Y/PY to 5.2 or 5.3: Are the proportion of participants and reasons for missing data similar across interventions? | NA |
|  |  |  |
|  |  |  |
|  | 5.5 If PN/N to 5.1, or Y/PY to 5.2 or 5.3: Is there evidence that results were robust to the presence of missing data? | NA |
|  |  |  |
|  |  |  |
|  | **Risk of bias judgement** | **Low** |
| Bias in measurement  of outcomes | 6.1 Could the outcome measure have been influenced by knowledge of the intervention received? | N |
|  |  |  |
|  | 6.2 Were outcome assessors aware of the intervention received by study participants? | Y |
|  |  |  |
|  | 6.3 Were the methods of outcome assessment comparable across intervention groups? | PY |
|  |  |  |
|  | 6.4 Were any systematic errors in measurement of the outcome related to intervention received? | N |
|  |  |  |
|  | **Risk of bias judgement** | **Low** |
| Bias in selection of the reported result | 7.1 multiple outcome measurements within the outcome domain? | N |
|  |  |  |
|  | 7.2 multiple analyses of the intervention outcome relationship? | N |
|  |  |  |
|  | 7.3 different subgroups? | N |
|  |  |  |
|  | **Risk of bias judgement** | **Moderate** |
|  | **Overall bias** | **Moderate** |
| Y: Yes; PY: Probably Yes; N: NO; NA: Not Applicable | | |
| **Fleming 2011** |  |  |
| Bias domain | Signaling question | Response options |
| Bias due to confounding | 1.1 Is there potential for confounding of the  effect of intervention in this study? | PY |
|  |  |  |
|  | 1.2. Was the analysis based on splitting participants’ follow up time according to intervention received? | N |
|  |  |  |
|  | 1.3. Were intervention discontinuations or switches likely to be related to factors that are prognostic for the outcome? | NA |
|  |  |  |
|  | 1.4. Did the authors use an appropriate analysis method that  controlled for all the important confounding domains? | Y |
|  |  |  |
|  | 1.5. If Y/PY to 1.4: Were confounding domains that were controlled for measured validly and reliably by the variables available in this study? | Y |
|  |  |  |
|  |  |  |
|  | 1.6. Did the authors control for any post-intervention variables that could have been affected by the intervention? | Y |
|  |  |  |
|  | 1.7. Did the authors use an appropriate analysis method that  adjusted for all the important confounding domains and for time varying confounding? | NA |
|  |  |  |
|  |  |  |
|  | 1.8. If Y/PY to 1.7: Were confounding domains that were adjusted for measured validly and reliably by the variables available in this study? | NA |
|  |  |  |
|  |  |  |
|  | **Risk of bias judgement** | **Moderate** |
| Bias in selection of participants into the study | 2.1. Was selection of participants into the study (or into the analysis) based on participant characteristics observed after the start of intervention? | N |
|  |  |  |
|  |  |  |
|  | 2.2. If Y/PY to 2.1: Were the post intervention variables that influenced selection likely to be associated with intervention? | NA |
|  |  |  |
|  | 2.3 If Y/PY to 2.2: Were the post intervention variables that influenced selection likely to be influenced by the outcome or a cause of the  outcome? | NA |
|  |  |  |
|  |  |  |
|  | 2.4. Do start of follow-up and start of  intervention coincide for most participants? | PY |
|  |  |  |
|  | 2.5. If Y/PY to 2.2 and 2.3, or N/PN to 2.4: Were adjustment techniques used that are likely to correct for the presence of selection biases? | NA |
|  |  |  |
|  |  |  |
|  | **Risk of bias judgement** | **Low** |
| Bias in classification of interventions | 3.1 Were intervention groups clearly defined? | Y |
|  |  |  |
|  | 3.2 Was the information used to define intervention groups recorded at the start of the intervention? | Y |
|  |  |  |
|  | 3.3 Could classification of intervention status have been affected by knowledge of the outcome or risk of the outcome? | N |
|  |  |  |
|  |  |  |
|  | **Risk of bias judgement** | **Low** |
| Bias due to deviations from intended interventions | 4.1. Were there deviations from the intended intervention beyond what would be expected in usual practice? | NA |
|  |  |  |
|  | 4.2. If Y/PY to 4.1: Were these deviations from intended intervention unbalanced between groups and likely to have affected the outcome? | NA |
|  |  |  |
|  |  |  |
|  | 4.3. Were important co-interventions balanced across intervention groups? | Y |
|  |  |  |
|  | 4.4. Was the intervention implemented successfully for most participants? | Y |
|  |  |  |
|  | 4.5. Did study participants adhere to the assigned intervention regimen? | Y |
|  |  |  |
|  | 4.6. If N/PN to 4.3, 4.4 or 4.5: Was an appropriate analysis used to estimate the effect of starting and adhering to the  intervention? | NA |
|  |  |  |
|  |  |  |
|  | **Risk of bias judgement** | **Low** |
| Bias due to missing data | 5.1 Were outcome data available for all, or nearly all, participants? | Y |
|  |  |  |
|  | 5.2 Were participants excluded due to missing data on intervention status? | N |
|  |  |  |
|  | 5.3 Were participants excluded due to missing data on other variables needed for the analysis? | N |
|  |  |  |
|  | 5.4 If PN/N to 5.1, or Y/PY to 5.2 or 5.3: Are the proportion of participants and reasons for missing data similar across interventions? | NA |
|  |  |  |
|  |  |  |
|  | 5.5 If PN/N to 5.1, or Y/PY to 5.2 or 5.3: Is there evidence that results were robust to the presence of missing data? | NA |
|  |  |  |
|  |  |  |
|  | **Risk of bias judgement** | **Low** |
| Bias in measurement  of outcomes | 6.1 Could the outcome measure have been influenced by knowledge of the intervention received? | N |
|  |  |  |
|  | 6.2 Were outcome assessors aware of the intervention received by study participants? | Y |
|  |  |  |
|  | 6.3 Were the methods of outcome assessment comparable across intervention groups? | PY |
|  |  |  |
|  | 6.4 Were any systematic errors in measurement of the outcome related to intervention received? | N |
|  |  |  |
|  | **Risk of bias judgement** | **Moderate** |
| Bias in selection of the reported result | 7.1 multiple outcome measurements within the outcome domain? | N |
|  |  |  |
|  | 7.2 multiple analyses of the intervention outcome relationship? | N |
|  |  |  |
|  | 7.3 different subgroups? | N |
|  |  |  |
|  | **Risk of bias judgement** | **Moderate** |
|  | **Overall bias** | **Moderate** |
| Y: Yes; PY: Probably Yes; N: NO; NA: Not Applicable | | |
|  |  |  |
